# Biocomposite nanofiber matrices to support ECM remodeling by human dermal progenitors and enhanced wound closure

**DOI:** 10.1038/s41598-017-10735-x

**Published:** 2017-08-31

**Authors:** Fraz Anjum, Natacha A. Agabalyan, Holly D. Sparks, Nicole L. Rosin, Michael S. Kallos, Jeff Biernaskie

**Affiliations:** 1 Pharmaceutical Production Research Facility, University of Calgary, 2500 University Drive N.W., Calgary, T2N 4N1 AB Canada; 2 Department of Comparative Biology and Experimental Medicine, Faculty of Veterinary Medicine, University of Calgary, 3300 Hospital Drive N.W., Calgary, T2N 4N1 AB Canada; 3 Department of Chemical and Petroleum Engineering, Schulich School of Engineering, University of Calgary, 2500 University Drive N.W., Calgary, T2N 4N1 AB Canada; 4 Department of Surgery, Cumming School of Medicine, University of Calgary, 3300 Hospital Drive N.W., Calgary, T2N 4N1 AB Canada; 5 Alberta Children’s Hospital Research Institute, University of Calgary, 3300 Hospital Drive N.W., Calgary, TN1 4N1 AB Canada; 6 Hotchkiss Brain Institute, University of Calgary, 3300 Hospital Drive N.W., Calgary, T2N 4N1 AB Canada

## Abstract

Cell-based therapies have recently been the focus of much research to enhance skin wound healing. An important challenge will be to develop vehicles for cell delivery that promote survival and uniform distribution of cells across the wound bed. These systems should be stiff enough to facilitate handling, whilst soft enough to limit damage to newly synthesized wound tissue and minimize patient discomfort. Herein, we developed several novel modifiable nanofibre scaffolds comprised of Poly (ε-caprolactone) (PCL) and gelatin (GE). We asked whether they could be used as a functional receptacle for adult human Skin-derived Precursor Cells (hSKPs) and how naked scaffolds impact endogenous skin wound healing. PCL and GE were electrospun in a single facile solvent to create composite scaffolds and displayed unique morphological and mechanical properties. After seeding with adult hSKPs, deposition of extracellular matrix proteins and sulphated glycosaminoglycans was found to be enhanced in composite grafts. Moreover, composite scaffolds exhibited significantly higher cell proliferation, greater cell spreading and integration within the nanofiber mats. Transplantation of acellular scaffolds into wounds revealed scaffolds exhibited improvement in dermal-epidermal thickness, axonal density and collagen deposition. These results demonstrate that PCL-based nanofiber scaffolds show promise as a cell delivery system for wound healing.

## Introduction

Besides providing a physical barrier that prevents pathologic infection, skin also performs a range of vital functions that maintain hydration, thermoregulation and body metabolism. Severe skin injury, such as burns and chronic non-healing wounds, result in lifelong functional impairment and, due to their need for chronic medical care, represent a substantial burden on healthcare. It is estimated that there are 6.5 million patients with chronic wounds in the United States alone, costing US$25 billion annually^1^. In particular, full thickness burns, in which both the epidermal and dermal compartments are damaged, are unable to repair to their full capacity^2^. Mammalian wound healing has evolved in favor of rapid closure, resulting in dysfunctional fibrotic scars. One promising approach to promote regeneration whilst minimizing scar is to engineer the local environment to promote coordinated cellular infiltration, organized deposition of extracellular matrix (ECM) and to provide instructive cues to promote regeneration of neodermis and appendage formation when combined with competent dermal cells.

An ideal cell scaffold should resemble the native extracellular matrix and be capable of supporting cell adhesion, proliferation and maturation. Electrospun mats have the potential to mimic the dermal ECM and can be tailored to encompass appropriate porosity, pore size, high surface to volume ratio, gas permeability and mechanical integrity, further supporting their utility for tissue engineering^3, 4^. Poly(ε-caprolactone) (PCL) is an FDA-approved semicrystalline biodegradable polyester used in a number of medical and drug delivery devices, including suture material^5, 6^ as it has excellent blend compatibility with various materials. Furthermore, the sole degradation product of PCL is caproic acid, a non-toxic metabolite which is either metabolized via citric acid cycle or eliminated via urinary secretion^7^. However, due to a lack of cell-recognition sites and poor hydrophilic character, PCL has shown reduced cell adhesion, proliferation and migration when utilized for a biologic scaffold based cell delivery system^8^. Conversely, gelatin (GE) is a protein-based biopolymer obtained by the partial hydrolysis of collagen. By virtue of its inherit biodegradability, biocompatibility and low immunogenicity, as well as its low cost and commercial availability, it has been widely used in skin tissue engineering^9, 10^ and wound healing dressings^11–13^. Gelatin is also employed in combination with many natural and synthetic materials to make sponges^14^, films^15^ and nanofibers^16^ for treating various forms of skin wounds. However, as gelatin alone has poor tensile strength, we blended (PCL-bGE) and coated (PCL-cGE) PCL with gelatin in the current study to achieve more desirable handling characteristics and asked whether PCL-GE composite nanofibers could provide the basis for a future cell-instructive scaffold for improved skin wound healing.

Cell adhesion and retention within the scaffold are of vital importance in tissue engineering and much work has been done functionalizing biopolymers to obtain a specific cell surface interaction. An equally important consideration in biomaterial design and development is the interaction of the material *in vivo*, as it could lead to foreign body reactions including inflammation and infection, as well as thrombosis and embolization^17^. The most common approach to improve biomaterials is to immobilize the material with cell recognition sequences to obtain controlled cell interaction with synthetic substrates^18–22^. The RGD (arginine-glycine-aspartate) motif is present in many ECM proteins and cell adhesion to this sequence is mediated by the interaction of surface receptors (integrins present in the cell membrane) to ligands bound to ECM proteins^23^. Polymers conjugated with RGD often exhibit a dramatic increase in cell adhesion and proliferation, as described with fibroblasts^24^ and osteoblasts^25^. Thus, in the current study, we investigated if a PCL-RGD conjugated polymer would improve performance of the scaffold.

Important steps towards the formation of skin substitutes have been made using differentiated cell types (such as endothelial cells, fibroblasts and keratinocytes)^26^. However, skin regeneration based on a tissue resident stem cell has not been explored in depth and hence provides a promising avenue for burn and wound healing. To recreate functional skin tissue within wounds (particularly for large or chronic wounds), scaffolds will need to be seeded with an abundant and accessible cell source, ideally from the patient’s own tissues to minimize concerns related to immunogenicity and disease transmission. Most importantly, donor cells should harbor the capacity to regenerate new dermal tissue themselves. Autologous bone marrow-derived mesenchymal stem cells (MSCs), lacking the ethical issues and safety concerns of embryonic stem cells (ESCs), have shown promise in modulating wound repair^27^. However, the invasive isolation procedure carries a high risk for individuals with severe burn disease or systemic illness leading to chronic wounds^28^. Alternatively, a population of dermal stem cells residing in hair follicles (hfDSCs) has recently been identified which function to continuously generate new dermal fibroblasts that populate the inductive dermal papilla and connective tissue sheath^29^. hfDSCs can be prospectively isolated from rodent skin^29, 30^ and phenotypically similar dermal progenitors can be isolated from adult human skin where they form self renewing colonies (referred to as skin-derived precursors or ‘SKPs’) *in vitro* ^31^. It has been demonstrated that following transplantation into injured skin or in combination with competent epithelial cells, rodent hfDSCs have the capacity to generate new dermal cells as well as skin appendages, both of which are important for the functional restoration of skin following injury^31^. As these hSKPs/dermal progenitors can readily be propagated from a minimally-invasive skin biopsy and can generate a diverse array of dermal fibroblast phenotypes, they hold great promise as a renewable source of progenitors for improved dermal wound healing.

To begin developing a nanofibrous biomaterial construct that could promote endogenous wound healing, whilst enabling directed delivery of dermal progenitors, we first examined the effect of various empty scaffolds each having different biophysical properties, on murine skin wound healing in *vivo*. In parallel, we investigated the impact of each construct on the behavior of seeded dermal progenitors in order to identify those materials that were most compatible as a delivery vehicle into wounds.

## Results

### Properties of PCL and PCL-Gelatin nanofiber mats

**Figure 1:**
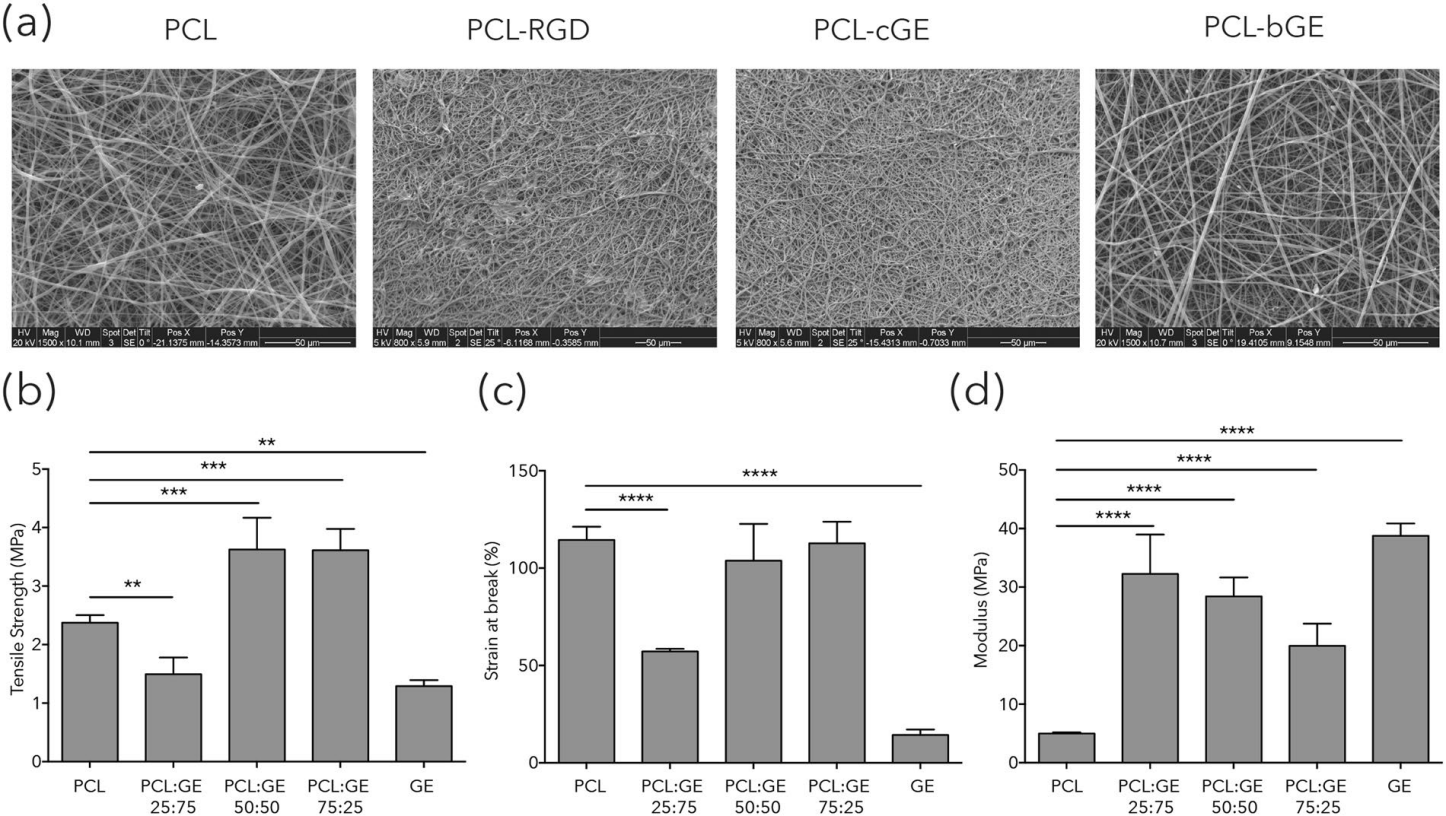
Properties of PCL and composite nanofiber mats. *Top*: SEM images of nanofibers. PCL 10% in CHCl_3_:MeOH (3:1), PCL immobilized with RGD (PCL-RGD), PCL-bGE (50:50) in TFE, PCL coated with gelatin (PCL-cGE). *Bottom*: Mechanical properties of PCL based nanofibrous scaffolds. (**a**) Tensile strength. (**b**) Strain at break (**c**) Young’s modulus. Data is presented as mean ± SEM for n = 3 for each group. **** represents p = 0.0001, *** represents p < 0.001 and ** represents p < 0.01.

The surface morphology of PCL-gelatin nanofibers was studied by SEM. Figure 1a shows SEM images of electrospun PCL and PCL-bGE composite (50:50) random nanofibers. SEM micrographs of PCL-cGE were also acquired to access the surface morphology of scaffolds for comparison purposes. Smooth, bead-free composite nanofibers were obtained at 15 kV applied voltage and 20 cm distance between needle tip and collector at a concentration of 10% for PCL and 12% for PCL-bGE composite fibers. In agreement with published reports^32^, the fiber morphology depends on the weight ratio of PCL to GE. Increasing the concentration of gelatin in the blend decreased the fiber diameter as well as the smoothness. The average fiber diameter of nanofibers was 797 ± 173 nm, 711 ± 157 nm and 666 ± 164 nm with 25, 50 and 75 wt% of gelatin respectively (Table 1). Porosity is also an important parameter while selecting scaffolds for wound healing to enhance cellular ingrowth and infiltration of nutrients. Favorable porosity values for tissue engineering scaffolds is between 60–90%^33^. The porosities of all composite and PCL-gelatin nanofibers were all approximately 80% and did not differ significantly from each other (Table 1). The pore diameter of PCL-bGE composite nanofiber mats was decreased with increasing the wt% of gelatin in the blend, that is 3.45 ± 0.65 µm (25 wt% gelatin) and 2.27 ± 0.61 µm (75 wt% gelatin).

**Table 1:** Properties of PCL and PCL-bGE composite nanofibers.

	Fiber Diameter (nm)	Pore size (µm)	Porosity (%)
PCL	563 ± 188	2.55 ± 0.58	80.68 ± 2.94
PCL-bGE 75:25	797 ± 173	3.45 ± 0.65	82.95 ± 0.99
PCL-bGE 50:50	711 ± 157	2.91 ± 0.60	83.18 ± 3.4
PCL-bGE 25:75	666 ± 164	2.27 ± 0.61	81.35 ± 3.02
GE	653 ± 144	3.02 ± 0.44	82.96 ± 5.49

Differential scanning calorimetry (DSC) was employed to assess the phase miscibility of PCL-GE composite nanofibers. Figure S1 represents the thermogram of the nanofiber meshes used in this study. PCL nanofibers showed an endothermic peak at 55.4 °C which is the melting temperature (*T*_*m*_) of PCL, while pure gelatin nanofiber meshes showed a peak at 98.3 °C which is the denaturation temperature (*T*_*d*_) of gelatin related to its helix-coil transition^34^. In the DSC thermograms of PCL-GE composite nanofibers composed primarily of PCL (50:50 and 75:25), the characteristic peak of gelatin denaturation disappeared, demonstrating the miscible morphology of nanofibers; while in nanofibers composed mainly of gelatin (25:75), the appearance of both characteristic peaks indicated the coexistence and phase separation of PCL and gelatin in composite nanofibers. However, the melting peak of PCL was lowered by 1–1.5 °C in composite fibers.

Mechanical evaluation of composite nanofiber membranes revealed that nanofiber meshes blended with 50 and 75 wt% of gelatin showed maximum tensile strength (3.6 ± 0.5 MPa, p = 0.0002 compared to PCL alone), while 25 wt% of gelatin and pure gelatin nanofiber sheets showed minimum tensile strength (p = 0.0044 and p = 0.0011 respectively compared to PCL alone) (Fig. 1b). However, the Young’s modulus of PCL-GE composite nanofiber mats was intermediate between the extremes of PCL (4.9 ± 0.2 MPa) and gelatin (38.7 ± 2.1 MPa, p = 0.0001) (Fig. 1d). Similar behavior was observed for the elongation properties of composite nanofibers sheets (Fig. 1c, p = 0.0001). Although the concentration of gelatin in the composite scaffolds determines the physical and biological properties of the resulting nanofibers, the nanofiber meshes obtained by blending 50 wt% of gelatin showed appropriate porosity as well as enough mechanical integrity to bear stresses during grafting procedures *in vivo*. Therefore, a 50:50 ratio was chosen for the PCL-bGE nanofiber sheets for the following *in vitro* as well as *in vivo* studies.

In the present study, PCL was immobilized with GRGDS via aminolysis as well as blended with gelatin with the aim of developing biologically inspired biocomposite matrices. Amino acid analysis, determined by Ninhydrin staining (Figure S2), revealed that the amount of GRGDS immobilized onto PCL-RGD nanofiber membranes was 0.021 ± 0.0019 µg/mg of scaffolds.

### Wound closure rate analysis

**Figure 2:**
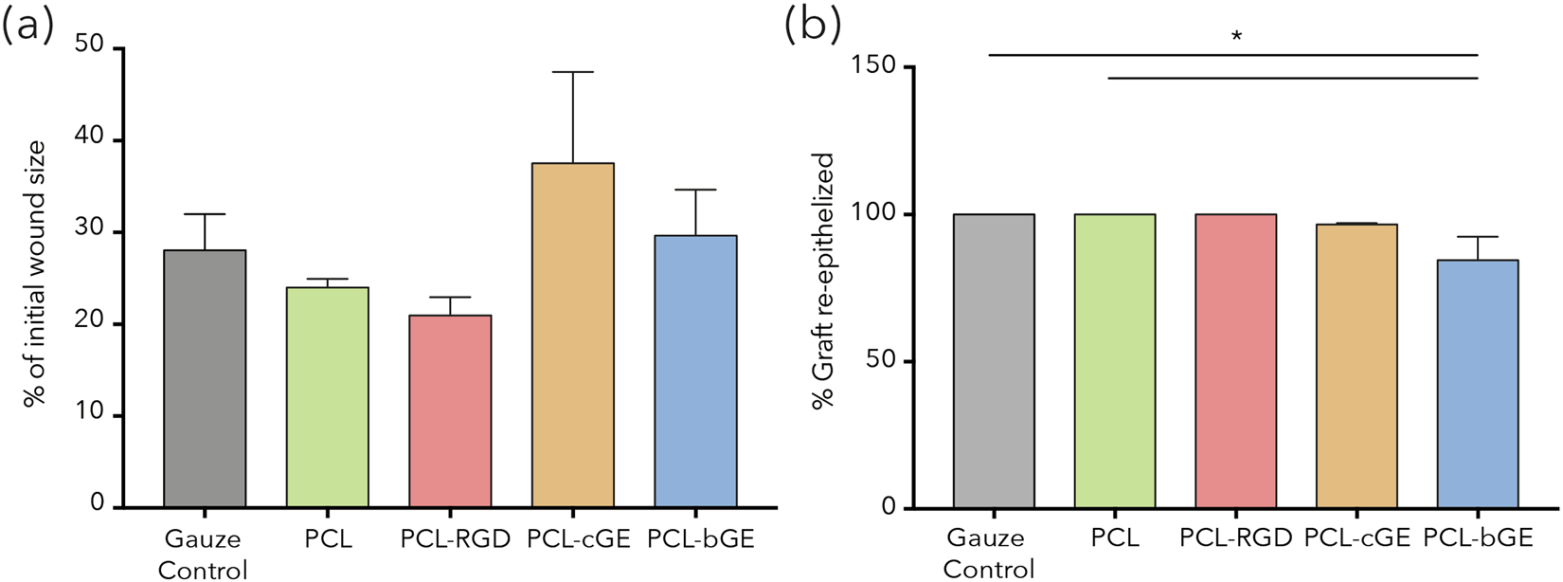
Wound closure analysis of PCL based scaffolds. (**a**) Analysis of wound closure rates of different PCL based scaffolds shows lesser contraction of PCL-cGE and PCL-bGE scaffolds. (**b**) Analysis of re-epithelization of wounds shows most scaffolds support re-epithelization, except PCL-bGE which was significantly lower. Data is presented as mean ± SEM for n = 3 for each group. * represents p < 0.05.

We then asked whether acellular nanofiber scaffolds, when placed in the wound bed, could support cell migration leading to tissue repair and regeneration and ultimately enhance the rate of wound healing by grafting each of the four scaffold types into surgically created wounds in immunocompromised mice (Figure S3). Measurement of wound area over three weeks revealed no significant difference in rate of closure between the various scaffolds versus gauze control or PCL was observed (Fig. 2a). After 21 days, the percent re-epithelization was analyzed histologically (Fig. 2b) revealing that all groups exhibited complete re-epithelization (Fig. 2b) with the exception of PCL-bGE re-epithelization which appeared to be delayed relative to the gauze control (p = 0.0217) and PCL alone (p = 0.0217).

### Dermal and epidermal thickness analysis

**Figure 3:**
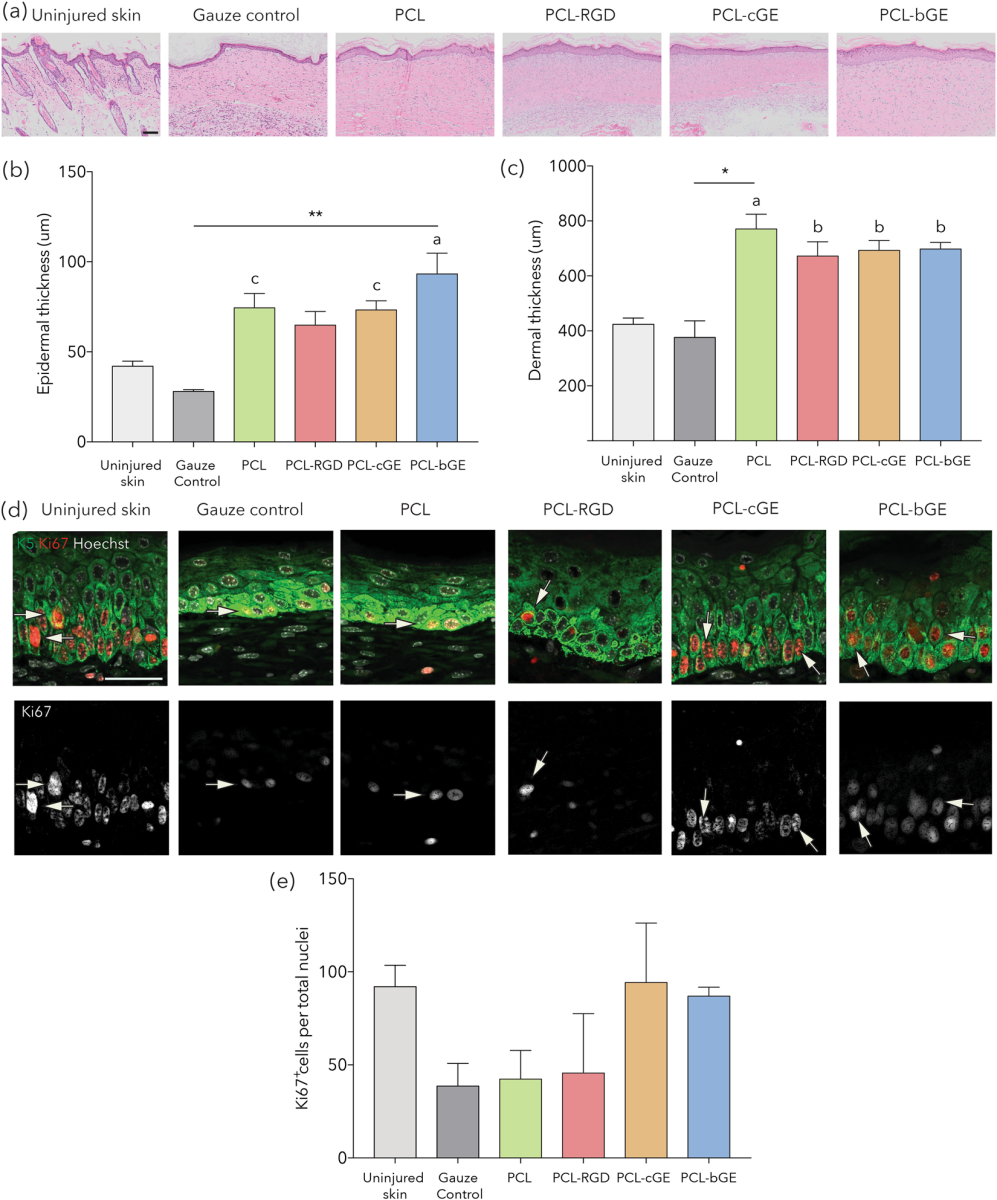
Histological analysis of skin ultrastructure and epidermal/dermal thickness following wound healing. (**a**) Representative images of hematoxylin and eosin staining of the grafts. (**b**) Analysis of epidermal thickness shows an elevated thickness in all groups compared to control (asterisks), including a significant increase in thickness for the PCL-bGE scaffold. PCL, PCL-cGE and PCL-bGE show a significant difference to uninjured skin (letters). (**c**) Analysis of dermal thickness shows an elevated thickness in all scaffolds compared to control (asterisks), including a significant increase for the PCL alone scaffold. All groups show a significant difference to uninjured skin (letters). (**d**) Representative merged images of keratin 5 (green), Ki67 (red) and nuclei (white) and separate images of Ki67 (white) immunolabelling (white arrows) in the epidermal layers. (**e**) Analysis of Ki67^+^cells per mm in the epidermal basal layer shows no significant differences between scaffolds. Bar = 100 μm. Data is presented as mean ± SEM for n = 3 for each group. **represents p < 0.01 and * represents p < 0.05. a represents p < 0.001, b represents p < 0.01 and c represents p < 0.05.

To assess dermal and epidermal thicknesses post healing, hematoxylin and eosin staining was performed (Fig. 3a) and three measurements of dermal and epidermal thickness were taken at equal distances along the length of the graft (on each of four sections per graft). PCL-bGE grafts were found to have a significantly thicker epidermis than the gauze control (p = 0.0086 - Fig. 3b), however none of the composite grafts were different from PCL alone. PCL, PCL-cGE and PCL-bGE remained significantly different from uninjured skin (p = 0.0248, p = 0.0309 and p = 0.0010 respectively). Only PCL grafts showed a significant increase in dermal thickness compared to the gauze control when implanted *in vivo* (p = 0.0233), however an increase in dermal thickness in all grafted groups was noted compared to the gauze control and all were significantly different to uninjured skin (p = 0.0005, p = 0.0073, p = 0.0041, p = 0.0036) (Fig. 3c). Proliferation in the epidermis was assessed by immunohistochemical staining of Ki67 and showed proliferating cells in the epidermis of all of the scaffold containing samples (Fig. 3d). There were no significant differences in the number of proliferating cells in the epidermis of all scaffolds (Fig. 3e) suggesting that the grafts did not impair epidermal basal layer function.

### Innervation and vascularization

**Figure 4:**
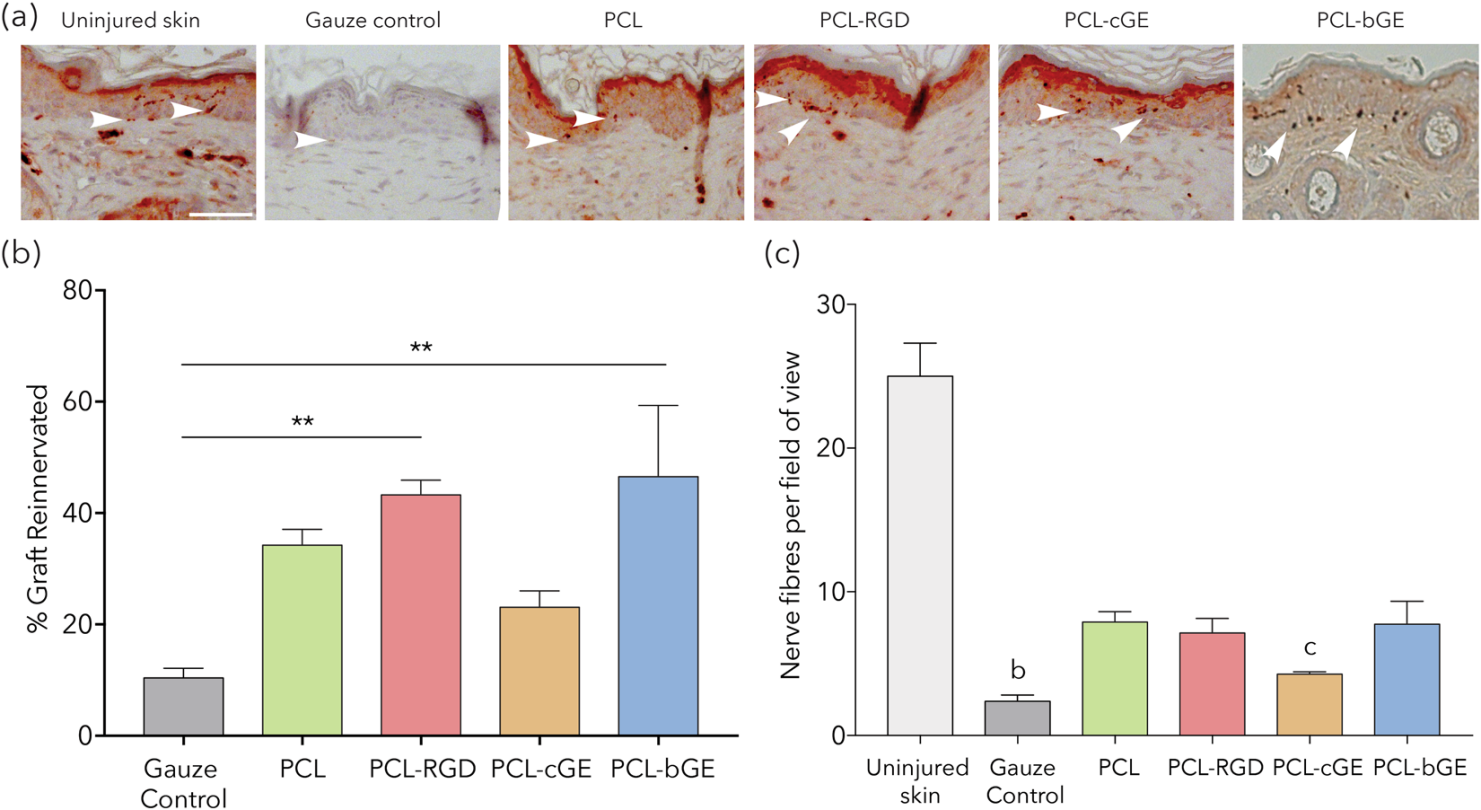
Immunohistochemical analysis of innervation and vascularization of healing skin. (**a**) Representative images of AEC^+^ chromagen βIII tubulin (dark red dots – white arrows) staining of the grafts. (**b**) Analysis of the total length of the graft re-innervated expressed as % shows a significant increase in PCL-RGD and PCL-bGE scaffolds compared to gauze control (asterisks). (**c**) Analysis of total number of nerve fibers crossing basal layer of epidermis shows a significant reduction from uninjured skin (letters) but no significant differences between scaffolds. Bar = 100μm. Data is presented as mean ± SEM for n = 3 for each group. ** represents p < 0.01. b represents p < 0.01 and c represents p < 0.05.

Immunohistochemical staining for βIII tubulin (Fig. 4a) revealed PCL-RGD and PCL-bGE grafts to have nerve fibers re-innervating a significantly greater length of the wounded area than the gauze control (p = 0.0139 and p = 0.0423, respectively). No significant difference was found between composite scaffolds when compared to PCL scaffold alone (Fig. 4b). Analysis of the density of nerve fibers crossing the basal layer at the graft margin alone did not reveal any significant differences between the scaffolds and the gauze control nor between composite scaffolds and PCL alone. However, all showed very reduced numbers of nerve fibers when compared to normal uninjured skin, with gauze control and PCL-cGE being significantly different to uninjured skin (p = 0.0029 and p = 0.0294 respectively) (Fig. 4c). We also performed immunostaining for CD31 followed by density measures to assess skin vascularity, but we failed to find any significant differences between graft types, or when compared to the gauze control or uninjured skin (p > 0.05). This is not surprising as mouse wounds tend to vascularize well following excision injury, but demonstrates that none of the scaffolds impeded formation of new blood vessels within the wound bed.

### Collagen III analysis within the wound bed

**Figure 5:**
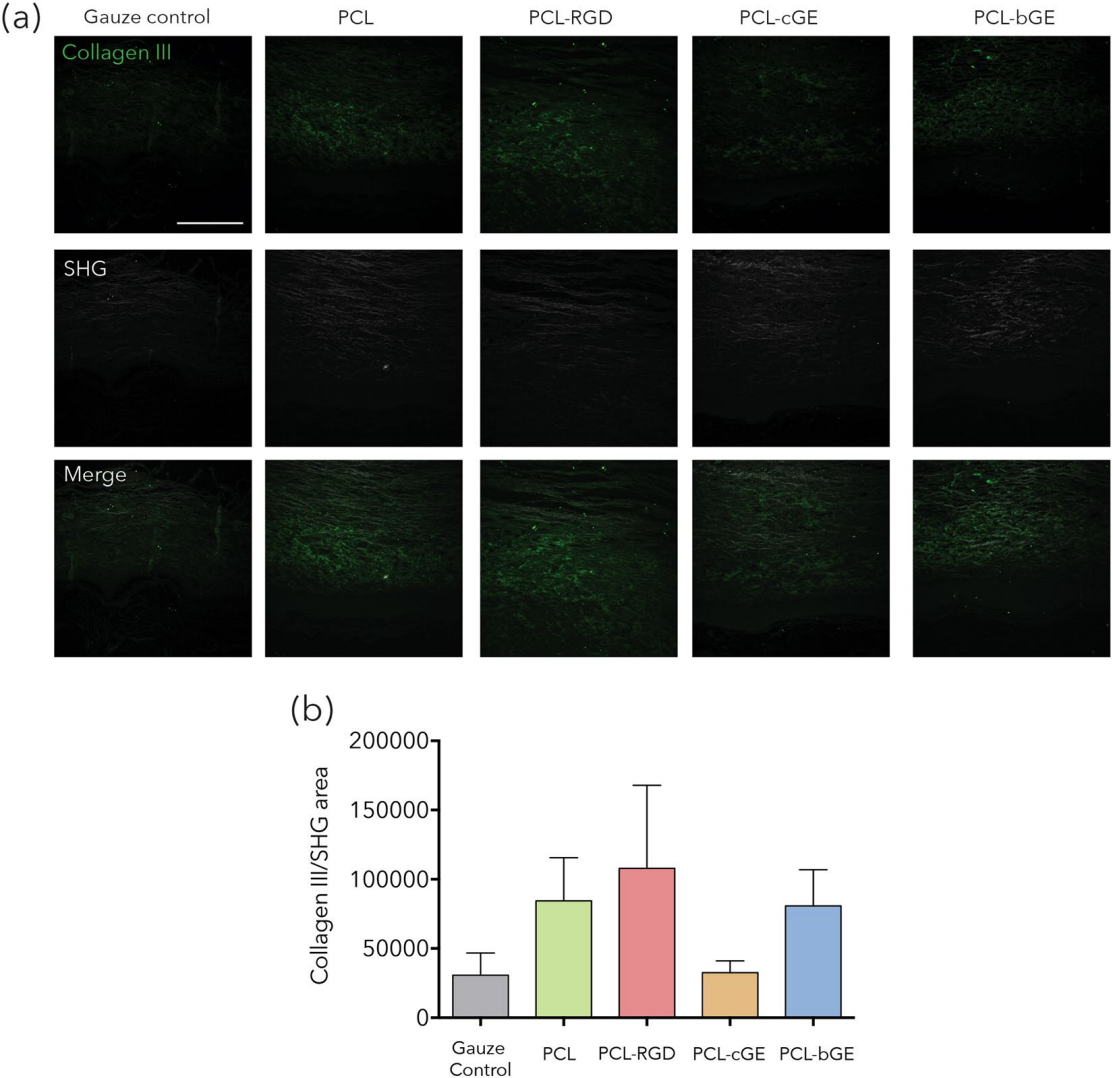
Second harmonic generation and immunohistochemical imaging of collagen content in grafted skin. (**a**) Representative images of collagen III staining (in green) overlapped with SHG imaging (white) of the grafts. (**b**) Analysis of the collagen III ratio per area shows no significant differences between scaffolds but a noted increase in the PCL-RGD scaffold. Bar = 100 μm. Data is presented as mean ± SEM for n = 3 for each group.

We used second harmonic generation (SHG), to specifically visualize fibrillar collagen deposition and organization. Combining SHG imaging with immunolabelling of Collagen III (Fig. 5a) revealed more collagen III in PCL, PCL-RGD and PCL-bGE groups than the gauze control. No significant differences were, however, found in overall fibrillar collagen as identified by SHG between the groups (Fig. 5b).

### Viability and proliferation of hSKPs

**Figure 6:**
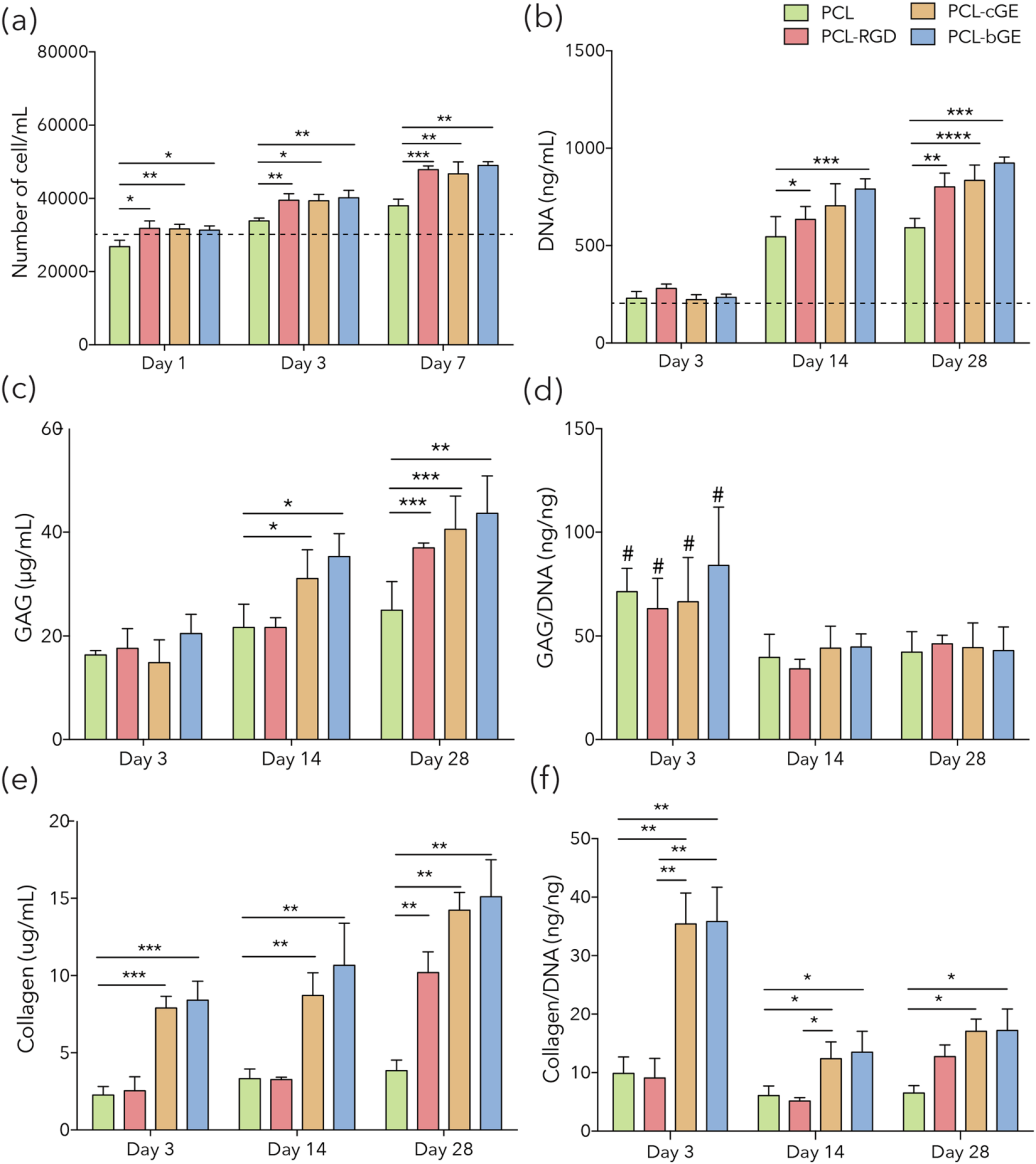
ECM components produced by hSKPs. (**a**) Cell proliferation determined by trypan blue at 1, 3 and 7 days. The initial cell density at day 0 was 30,000 cells/gel as indicated by the dotted line. (**b**) DNA content at 3, 14 and 28 days of hSKPs culture measured by CyQuant^TM^ showing the influence of RGD and gelatin on cell proliferation. The dotted line represents the DNA content immediately after seeding. (**c**) Glycosaminoglycan quantification of various nanofiber meshes at 3, 14 and 28 days determined by DMMB (**d**) GAG content normalized to total DNA content. # represents p < 0.05 which is statistically significant from day 14 and 28. (**e**) Total collagen content as measured by hydroxyproline calorimetric assay. (**f**) Collagen/DNA ratio. The data represents higher collagen and GAGs level on nanofiber scaffolds immobilized with RGD and composite with gelatin. Data is presented as mean ± SEM for n = 5 for each group. **** represents p = 0.0001, *** represents p < 0.001, ** represents p < 0.01 and * represents p < 0.05.

In order to determine whether these nanofiber scaffolds could be used as a vehicle for cell delivery into wounds, we examined the growth of hSKPs on four different scaffold compositions (PCL, PCL-RGD, PCL-cGE and PCL-bGE) *in vitro*. To assess cell viability in various conditions of PCL and PCL-GE nanofibrous mats, 30,000 hSKPs/scaffold were seeded and cultured in proliferation media for 1, 3 and 7 days before harvesting for cell counting. Figure 6a shows that 24 hrs post seeding, the cell density remained fairly constant but reached a peak cell density at day 7 with respect to initial cell density (all exact p-values in Table S1). This was confirmed by LIVE/DEAD viability assays at 1 and 7 day of culture showing spreading and proliferation of hSKPs (Figure S4).

To further evaluate cell proliferation, the DNA content of hSKPs seeded nanofiber mats was evaluated at 3, 14 and 28 days after seeding. For all conditions, DNA content increased by >3 fold between day 1 and day 28 (Fig. 6b). Scaffolds made with PCL-bGE showed maximum proliferation with a 4-fold increase in total DNA content, while PCL nanofibers showed an initial increase up to day 14, but then plateaued. Together this data shows that hSKPs remain viable and can proliferate upon seeding onto PCL and PCL-gelatin composite nanofiber membranes. A two-way ANOVA confirmed that PCL-RGD, PCL-cGE and PCL-bGE all showed significantly enhanced cell proliferation by cell counting up to 7 days of culture and DNA content following 4 weeks of growth on either substrate compared to PCL alone.

### ECM accumulation within nanofibrous scaffolds

ECM production is an important determinant of dermal fibroblast function. *In vivo*, rodent hfDSCs give rise to multiple fibroblast derivatives each producing distinct ECM profiles tailored to their local environment. To evaluate the influence of nanofiber composition on isolated dermal progenitor production of new ECM, glycosaminoglycans (GAGs) and total collagen content was quantified. Sulfated GAG content was quantified using a dimethyl methylene blue (DMMB) biochemical assay. At day 3, no differences were observed. However, at both 14 and 28 days of culture, PCL-cGE and PCL-bGE nanofiber scaffolds produced significantly higher GAG content than PCL alone (Fig. 6c). When GAG was normalized to DNA content, the ratio was high in all groups at day 3. However, it decreased at day 14 and remained constant in all groups until day 28 (Fig. 6d). As such, there was no statistically significant differences in normalized GAG/DNA among the various nanofiber meshes at later time points of culture (Fig. 6d). Collagen was quantified using a hydroxyproline colorimetric assay. At all time points, hSKPs seeded onto PCL-cGE and PCL-bGE scaffolds produced significantly higher total collagen content in comparison to PCL nanofibers. PCL-RGD nanofibers also produced higher total collagen content at 28 days post incubation (Fig. 6e). The ratio of collagen/DNA was significantly higher in PCL-cGE and PCL-bGE scaffolds than PCL alone at day 3 but decreased rapidly after 14 days. Although not statistically significant, the ratio again increases at 28 days of culture (Fig. 6f).

### Morphology and immunocytochemistry of hSKPs on nanofibrous scaffolds

**Figure 7:**
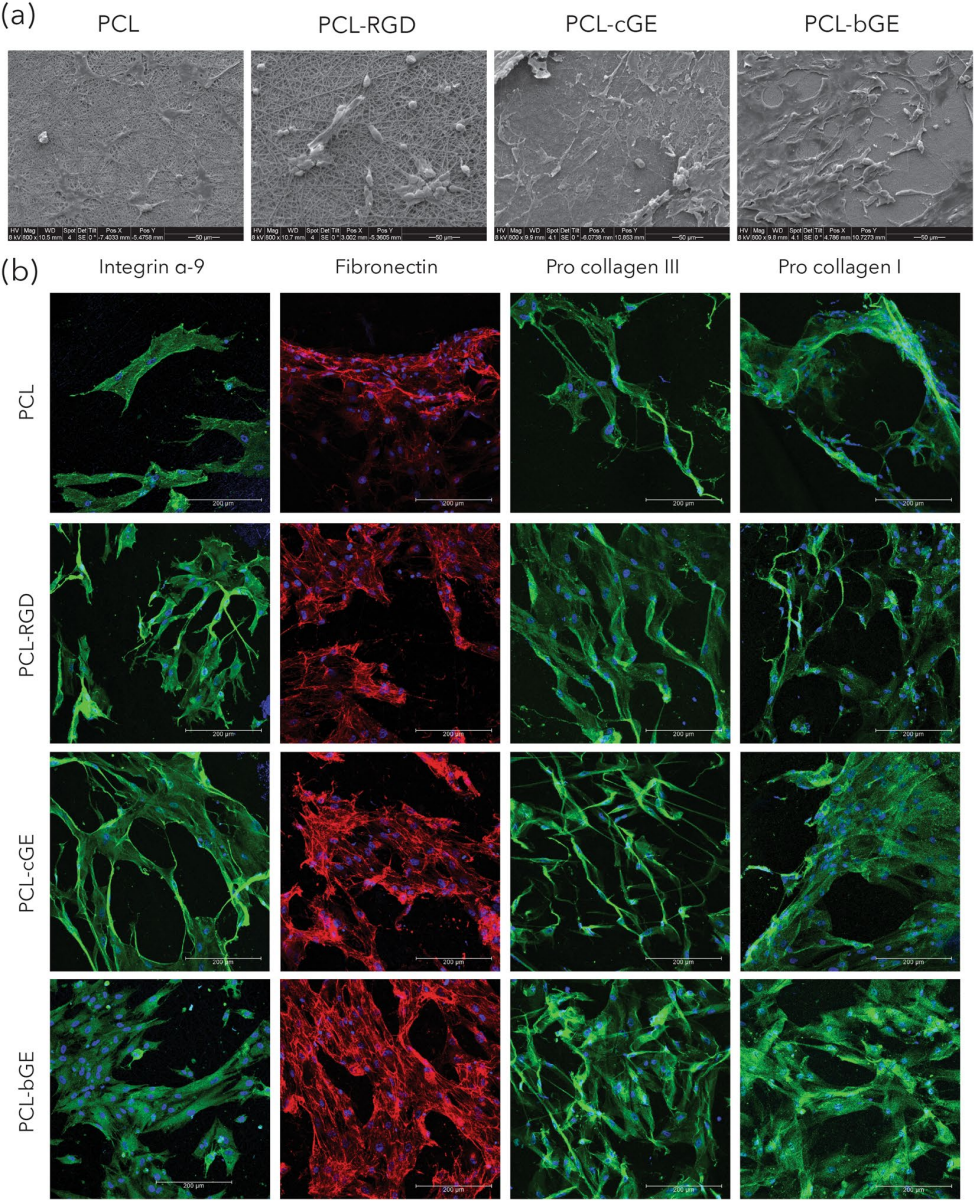
Morphology and protein expression of hSKPs. (**a**) SEM micrographs of nanofibrous scaffolds showing morphology of hSKPs at 7 days of culture. (**b**) Immunofluorescent images of hSKPs grown on nanofibrous scaffolds showing expression of integrin α-9 (green), fibronectin (red) and procollagen I & III (green).

SEM micrographs of the nanofiber meshes revealed that hSKPs begin to extend processes and cover the surface of nanofibers (Figs 7a and S5). By day 7, PCL-bGE nanofiber sheets were almost completely confluent with a monolayer of cells; the density of hSKPs on PCL fibers appeared lower than PCL-RGD, PCL-cGE and PCL-bGE composite scaffolds. To characterize the impact of various nanofiber mats on hSKP fate, cells were stained with several markers via immunocytochemistry. hSKPs continue to express integrin α-9, a marker expressed by dermal papilla cells^35^, fibronectin, a dermal fibroblast marker^31^ and fiber forming collagens I and III (synthesized as procollagen I and III), also highly expressed by fibroblasts^36^. Figure 7b shows fluorescent images of hSKPs stained with these markers. Seeded hSKPs show spreading morphology and were distributed evenly on PCL-cGE, PCL-bGE and PCL-RGD scaffolds. Cell elongation, spreading and integration within nanofiber mats were found to be superior with composite nanofiber scaffolds and scaffolds immobilized with RGD than PCL alone.

### Collagen analysis by SHG

**Figure 8:**
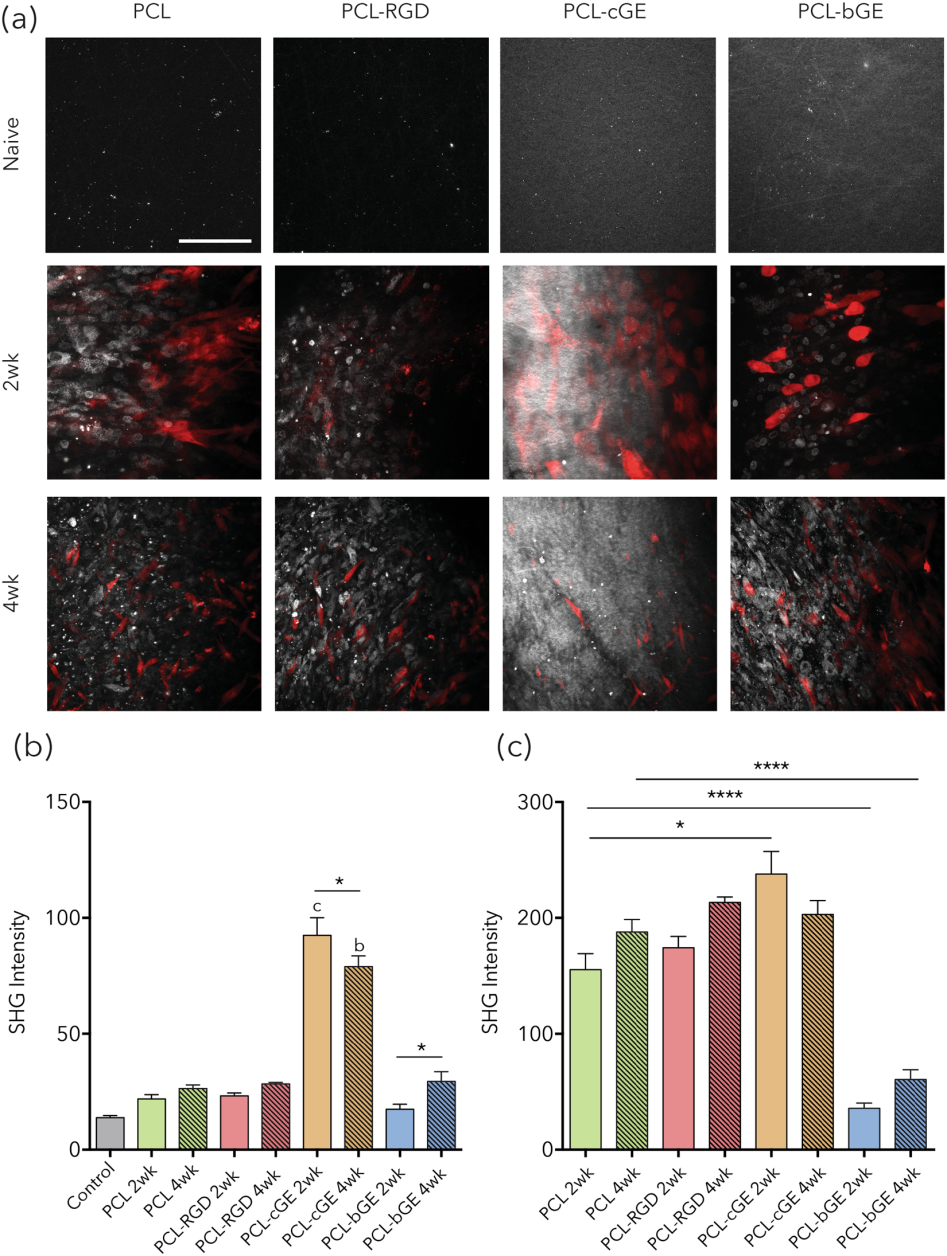
Second harmonic generation images of collagen deposited on nanofiber scaffolds seeded with TdTomato^+^ hSKP. (**a**) Representative SHG images of scaffolds without cells and with cells at 2 and 4 weeks in culture. SHG is represented by white and TdT^+^ cells in red. (**b**) Mean intensity of the SHG signal shows a decrease between 2 and 4 weeks for the PCL-cGE scaffold and an increase between 2 and 4 weeks for the PCL-bGE scaffold (asterisks). A significant increase at 2 and 4 weeks for the PCL-cGE compared to gauze control was also noted (letters). (**c**) Mean intensity of the SHG signal normalized to control shows a significant difference between PCL and PCL-cGE at 2 weeks and between PCL and PCL-bGE at both 2 and 4 weeks (asterisks). Bar = 100 μm. Data is presented as mean ± SEM for n = 3 for each group. **** represents p < 0.0001 and * represents p < 0.05. b represents p < 0.01 and c represents p < 0.05.

As an additional read-out of dermal repair within the wound, we used second harmonic generation (SHG), to specifically visualize fibrillar collagen (Types I and III) deposition and organization. The gelatin in both the coated and blended scaffolds was picked up by SHG imaging, however by 2wks in culture the hSKPs on all of the scaffold types appear to be producing collagen (Fig. 8a,b). There are no discernable differences between PCL, PCL-RGD and PCL-bGE composed nanofiber scaffolds, however PCL-cGE was significantly different to control at both 2 and 4 weeks (p = 0.0049 and p = 0.0029 respectively) (Fig. 8c). There was a significant decrease in PCL-cGE signal between 2 and 4 weeks (p = 0.0261) and a significant increase in PCL-bGE signal between 2 and 4 weeks (p = 0.0201). When normalized to the SHG intensity of the controls scaffolds, the collagen in PCL-cGE was significantly increased from PCL alone at 2 weeks (p = 0.0013) and PCL-bGE was significantly decreased from PCL alone at 2 and 4 weeks (p = 0001).

## Discussion

The present study was designed to explore the potential of nanofiber mats to enhance donor cell survival, proliferation, and production of ECM components, as well as the inherent capacity of acellular scaffolds to support endogenous wound healing in an *in vivo* mouse model. Our findings suggest that PCL based nanofibers are suitable to be used as a functional receptacle for adult hSKPs and show promise as a cell delivery system for wound healing.

Electrospun nanofiber scaffolds have been used as engineered skin substitutes due to the simple and cost-effective procedure of electrospinning. Herein, we used PCL and gelatin to produce nanofiber matrices. Due to the non-ionic nature of PCL, it is only soluble in organic solvents. Gelatin, however, is a polyelectrolyte possessing ionizable amino and carboxylate groups, thus producing ions upon dissolving in acidic or neutral solvents^37^. Moreover, proteins are least soluble at their isoelectric point (IP)^38^. Therefore, trifluoroethanol (TFE), which possesses a strong acidic character due to its high electronegative group, was used to dissolve both PCL and gelatin^39^. Pure gelatin is soluble at a concentration of 10 wt% in TFE; however, upon addition of PCL, the solubility of gelatin is reduced due to the increase in pH, thereby creating precipitates during the electrospinning process which cause defects in fiber morphology. To counteract this effect, a small amount of glacial acetic acid was added to the solution^39^ to control the pH of the mixture, thus keeping the solution miscible during electrospinning. In the composite polymer system, as the concentration of gelatin increases, a higher charge density is produced due to protein-protein interaction. An increasingly high charge density at the needle tip creates very high charge-charge repulsion causing a burst of droplets extruding out of the needle^40^. Here we have shown the concentration of gelatin in the blend directly affects fiber morphology as well as the ease of fiber formation.

Although PCL is shown to have a resorption time that ranges from several months to over a year, its degradation profile can be tailored by blending or copolymerization with other materials^41^. Further, PCL is also shown to be degraded enzymatically^42^. The second component of the blended nanofiber scaffolds, gelatin, is degraded by gelatinases and stromelysin^43^. These enzymes are secreted by various cells types including fibroblasts and epithelial cells during the inflammatory phase of wound healing and are shown to be involved in cell migration during the proliferative phase^44^. During our study, we observed all scaffolds were absorbed within the 21-day time frame, a parameter which merits to be tailored further to support enhanced repair of the ECM. Moreover, gradual solubility of gelatin under physiological conditions, deformation properties, and dynamic amoeboid movement exerted by cells can cause the widening of the pores as the culture progress^45^. These broadened pores help facilitate cell migration. Indeed, we observed cell migration deep into the scaffold, as evidenced by overlaying SHG and confocal imaging.

Ideally, scaffolds used for wound repair should have appropriate structural and mechanical integrity and elasticity so that they may be handled and grafted with ease by clinicians. Moreover, the scaffolds should support regulation of cellular activities and tissue repair through biomechanical stimuli, post implantation^46^. Commonly employed scaffolds for wound healing such as collagen and polylactide-co-glycolide have poor mechanical properties *in vivo* ^32, 47^. The pure gelatin nanofiber membranes used in this study showed only 14 ± 2.8% strain at break with tensile strength of 1.29 ± 0.1 MPa, in agreement with the published reports^39^. Therefore, it was not employed for *in vitro* and *in vivo* studies due to rapid solubility and handling constraints respectively. By blending gelatin with PCL (50:50), the elasticity was enhanced 7-fold to 103 ± 18% strain at failure with tensile strength of 3.6 ± 0.5 MPa. Therefore, our results showed that composite nanofiber scaffolds have sufficient mechanical strength and elasticity to bear the stresses endured during handling and grafting onto the wound site. Indeed, all the scaffolds used in this study showed strong enough mechanical integrity and elasticity to be handled during surgical implantation, including manual manipulation and suturing in place.

Repair of adult skin wounds often results in scar formation, and studies have suggested that some proteoglycans (PGs) are known to significantly reduce scar formation by ameliorating factors involved in fibrosis and fibroplasia as well as ECM deposition^33^. During wound healing, sulfated PGs levels are increased after 2 weeks of cell proliferative phase^34^ and help in collagen polymerization^48^. Furthermore, GAG-peptide fragments are released by protease activity on PG degradation in wounds, which help to modulate the wound healing process^49^. Sulfated GAGs also regulate growth factor activity which in turn modulate angiogenesis^50^. Overall GAGs play a key role in all phases of wound healing as well as in regulating ECM organization and metabolism^51^. Therefore, in addition to evaluating cellular proliferation via quantification of DNA, the effects of various PCL based nanofiber mats on ECM deposition were also studied. We noted an increased total DNA content of hSKP-seeded scaffolds over 28 days and observed PCL-RGD and PCL-gelatin composite scaffolds supported hSKPs culture better than PCL alone. From DMMB biochemical analysis, it was evident that PCL-gelatin composite scaffolds deposited significantly higher GAG and collagen at all time points during cell culture. In PCL-RGD scaffolds, the collagen production was delayed until day 14 after which it increased. Similar findings of up-regulation of collagen III were found with RGD immobilized alginate scaffolds^52^. This could be due to the fact that during initial *in vitro* culture, cells mostly adhere to non-homogeneous RGD binding sites until a confluent layer of cells is created. These results indicate that PCL scaffolds coated or blended with gelatin had a positive influence on the production of GAG and collagen by hSKPs up to 28 days of culture. Natural ECM is a nanoscale network of proteins and GAGs. Therefore, nanofiber meshes, due to their nanoporous network and close resemblance to endogenous architecture of ECM, provide optimal conditions for higher GAG and collagen production by cells which may ultimately lead to better cellular response. While both GAG and collagen content of hSKP-loaded nanofiber mats increased till day 28, GAG/DNA and collagen/DNA was highest at day 3, decreased at 14 days and remained fairly constant till day 28. This is due to the GAGs produced by cells being released into the surrounding medium during the culture.

Wound healing is a complex process involving 3 phases: inflammation, proliferation, and maturation^53^. In mice, wound healing is largely facilitated by contraction. During wound contraction, contractile fibroblasts in the dermis (myofibroblasts) work to close the wound by bringing the intact skin surrounding the wound together. In loose skinned mammals, like mice, this results in minimal scarring and rapid closure of the wound^54^. However, in humans and many other mammals, healing occurs mainly through the production of granulation tissue during the proliferative phase followed by re-epithelialization from the margins of the wound. This results in scar formation, characterized by an abnormal and excessive deposition of collagen as well as lack of important dermal appendages such as hair follicles and glands^55^. Due to the differences in healing response between mice and humans, we attempted to “splint” open the wound to prevent contraction by suturing the edges of the wound to the scaffold itself, or in the case of the control, an empty gauze^56^. Contraction was still observed in all wounds; however, central areas of the wound were observed to heal by granulation tissue formation and re-epithelialization in all groups. While it is essential wound closure is achieved within a certain time frame, it is thought the addition of a scaffold may contribute to better repair mechanisms in the wound.

An increase in epidermal and dermal thickness when compared to gauze control was found with all scaffolds. Our results suggest addition of a scaffold alone (PCL) shows improved healing when compared to no scaffold or gauze control) as this epidermal and dermal thickness could be an indication of the improved survival and/or proliferation of cells. The addition of gelatin and RGD sequences to the scaffold confirmed this effect, whilst also increasing cell survival and proliferation of hSKPs. Proliferating cells were found in the epidermis of all tested scaffold, with the PCL-GE combinations showing numbers closest to uninjured skin.

Although animal-derived acellular scaffolds (decellularized dermis) used for wound healing have been shown to enhance fibroblast infiltration and migration, clinical results have not always been satisfactory^57^. This is because most acellular scaffolds used for wound healing are crosslinked using chemicals which may be cytotoxic^58^. On the other hand, enhanced ECM deposition, greater cell infiltration, and improved neovascularization is evident in non-crosslinked matrices^58^. Our results showed improved re-innervation of the wounds in all scaffold groups, with PCL-RGD and PCL-bGE in particular showing results closest to uninjured skin. Collagen III production *in vivo* as assessed via immunostaining was found to be increased in all groups except PCL-cGE when compared to gauze control. SHG imaging revealed PCL-cGE to express significantly more collagen I than all other scaffolds when seeded with hSKPs; making it a less promising candidate for wound healing as it may be promoting a fibrotic response.

Isolated adult rodent dermal progenitors have been shown to improve skin wound healing; when transplanted into a full thickness wound, the cells were shown to repopulate the wound and contribute to the interfollicular dermis^31^. We hypothesize that SKPs isolated from adult human skin maintain this inherent regenerative capability and that transplantation of hSKPs may help improve skin repair following deep dermal wounds by contributing to the deposition of normal collagen, repopulating the dermal compartment. Given their inherent inductive function *in vivo* ^29, 31^, if a permissive environment could be achieved within the wound bed, it may be possible to promote regeneration of hair follicles and sebaceous glands. Indeed, appendage neogenesis has been demonstrated in adult mouse wounds, suggesting that true regeneration is possible within mammalian full-thickness skin wounds^59–61^. Supplementing wounds with additional inductive mesenchymal cells, in combination with a permissive substrate, could further augment this inherent regenerative response. However, the influence of SKPs on wound closure and dermal regeneration has not been evaluated in this study and warrants further investigation. In the current study, hSKPs cultured on nanofiber matrices expressed fibronectin, which is a crucial component of ECM shown to be involved in nerve fiber growth^62^. Other proteins expressed by hSKPs *in vitro* include collagens I and III, which are fiber forming, structural ECM proteins necessary for wound healing. Collagen production by hSKPs is indicative of their fibroblast-like phenotype after culture on nanofiber scaffolds. The collagen structure appears similar between all nanofiber scaffold compositions except for PCL-cGE, suggesting that gelatin coating is influencing hSKPs to either increase collagen production, increase collagen cross-linking or a combination of the two. Overall, this suggests that the integration of the hSKPs with nanofiber scaffolds will potentially enhance initial scaffold integration, and also participate in wound healing.

As previously described, electrospun nanofiber scaffolds provide a direct endogenous repair mechanism to guide intrinsic repair of lost tissue as well as support therapeutic delivery of cells to further improve wound healing. *In vivo* testing showed that PCL-based nanofiber scaffolds have the potential to improve wound healing in a full thickness murine skin wound model. In particular, PCL-bGE shows promise as a vehicle for hSKP transplantation. It showed increased GAG production, increased cell numbers, viability and *in vitro* growth of hSKPs and increased epidermal thickness. It appears to recapitulate uninjured skin the closest in terms of proliferation of basal epidermal cells and innervation of the epidermal layer. It also showed a high collagen III content compared to gauze and other scaffolds. PCL-RGD also showed these same characteristics, making it a secondary but also promising candidate for improved wound healing.

We have shown that non-crosslinked, cell-free biocomposite nanofiber membranes are appropriate inserts for improved skin repair following full thickness wounds. The scaffolds have been shown to support the attachment, growth and ECM remodeling of tissue resident stem cells (hSKPs) *in vitro* and improve the overall skin repair *in vivo*. In particular, the blended PCL-bGE nanofiber composite was shown to significantly increase viability and proliferation of hSKPs as well as to enhance production of ECM *in vitro* and within skin wounds *in vivo*. Overall, the results demonstrate that the present approach of non-crosslinked composite nanofiber scaffolds along with skin derived precursors may hold great promise for delivery of cells within injured tissues and specifically as a template for improved wound healing.

## Materials and Methods

### Nanofiber fabrication and characterization

#### Materials

Poly(ε-caprolactone) (cat #440744), Gelatin from bovine skin type B (cat #G9382), trifluoroethanol (TFE cat #T63002), *N,N*-Dimethylformamide (DMF cat #D4551)), dichloromethane (DCM cat 270997)), chloroform (cat #372978), 1,4-dioxane (cat #296309), glutaraldehyde (cat #G5882), Ninhydrin (cat #151171), 2-propanol (cat #I9516), sodium cyanoborohydride (cat #156159), acetic acid (cat #320099), 1,6-hexandiamine (cat #H11696), papain (cat #P4762), L-cysteine hydrochloride (cat #C1276) and dimethyl methylene blue (DMMB cat #341088) were all purchased from Sigma-Aldrich Canada and used as received. GRGDS peptide was obtained from Canpeptide Montreal Quebec.

#### Preparation of Electrospinning Solutions

A 10% w/v solution of poly(ε-caprolactone) (PCL, MW ~ 80,000) was prepared in chloroform:DMF (4:1) at room temperature. For the blend polymeric system, 10% w/v solution of PCL and 12% w/v solution of gelatin were prepared in trifluoroethanol (TFE) for 24 hrs at room temperature and the two polymer solutions were mixed at various proportions to create 3 different PCL:GE ratios (25:75, 50:50 and 75:25). A drop of glacial acetic acid was added to each polymer mixture to avoid phase separation during electrospinning^42^.

#### Fabrication of PCL and PCL-GE nanofiber meshes

The electrospinning was performed using an in-house electrospinning setup, equipped with a syringe, a blunt-tip metallic needle (Hamilton syringes Canada), a syringe pump (Model 11 Plus, Harvard Apparatus, Holliston MA) for controlling the feed rate of polymer solution, a high voltage DC power supply (Gamma High Voltage, Ormond Beach, FL) for charging the polymer solution and a metallic collector (flat plate or rotating drum) for collecting the nanofibers. The solution was placed in a 5cc syringe (Becton-Dickinson) with a 22-gauge blunt tip needle. The polymer solution was deposited on an aluminum plate collector at 0.4–1.2 mL/h and a voltage of 8–15 kV was applied. The fiber sheet was carefully peeled off the aluminum foil and placed in a vacuum desiccator until use.

#### Immobilization of GRGDS onto PCL nanofibers mats

PCL nanofiber meshes were immobilized with GRGDS peptide according to previously published reports^43^. Briefly, the nanofiber mats were immersed in 10% 1,6-hexandiamine in isopropanol for 1 h at 37 °C, rinsed with deionized water for 24 hrs to remove any free 1,6-hexandiamine and dried under a vacuum overnight. The aminolyzed PCL mats were immersed in a 1% glutaraldehyde solution for 3 hrs at room temperature followed by thorough rinsing with deionized water for 24 hrs to remove any free glutaraldehyde. The nanofiber mats were then immersed in a 5 mg/mL solution of GRGDS in PBS overnight at 4 °C and finally rinsed with deionized water and dried.

Determination of amino groups: To ensure appropriate manufacturing of the aminolysed PCL nanofiber mats (PCL-RGD), the mats were quantitatively measured via Ninhydrin analysis to measure the amount of NH_2_ group aminolysed onto the mats. The mats were immersed in 1 mol/L Ninhydrin in ethanol in a 50 mL glass petri dish for 1 min and then heated in a glass tube at 80 °C on oil bath for 15 min. After the evaporation of adsorbed ethanol, 5.0 mL of dioxane was added to the tube to dissolve the membrane. As the blue color appeared on the nanofiber surfaces an additional 5 mL of isopropanol was added to stabilize the blue compound. The absorbance was measured at 450–650 nm on a UV-Visible spectrophotometer. A calibration curve was established using a 1,6-hexandiamine in a dioxane:isopropanol mixture (1:1).

#### Characterization of Nanofibers

Morphology: Morphology of the electrospun PCL and PCL-GE fibers was observed using scanning electron microscopy (SEM) (Phillips, now FEI, XL30ESEM) operated at an acceleration voltage of 5–10 kV. Prior to imaging by SEM, samples were sputter-coated for 2 min with gold-platinum (Techniques Hummer I) to increase conductivity. Fiber diameters and pore size were measured using ImageJ software. SEM micrographs were first segmented using thresholding command of imageJ. The segmented images include only black and white pixels, where black representing background (pores) and white representing fibers. Analyze particles command of imageJ was then employed to measure the pore size (black background pixels). The mean pore size was then analyzed by processing a minimum of 5 SEM images in each condition. In order to measure fiber diameter, SEM images were zoomed in followed by scale calibration. The length of fiber diameter was measured by drawing a line command from one edge of fiber to the other. In case fiber dimeter is not uniform, three measurements were taken at three different places on the same fiber and took the mean value of a single fiber diameter. A minimum of 100 filaments of each sample from at least 5 SEM images were analyzed from random locations to get the average reported value of fiber diameter.

The apparent density and porosity of nanofibrous mats were calculated by using equations 1 and 2 respectively^44^.

1 $$\mathrm{Apparent}\,\mathrm{density}=\frac{mass\,of\,nanofiber\,mats\,(g)}{thickness\,of\,nanofiber\,mats\,(cm)\,\times \,area\,of\,nanofiber\,mats\,(c{m}^{2})}$$

2 $${\rm{Porosity}}=(1-\frac{apparent\,density\,of\,nanofiber\,mats\,(gc{m}^{-3})}{bulk\,denisty\,of\,nanofiber\,mats\,(gc{m}^{-3})})\times 100 \% $$

Fourier Transform Infrared Spectrophotometry (FT-IR): A Nexus 470 FTIR spectrometer equipped with a cesium iodide detector (GMI Inc. Ramsey MN) was used to obtain FTIR spectra of the electrospun PCL and PCL-bGE fibers over the range of 400–4000 cm^−1^ at a scanning resolution of 2 cm^−1^.

Mechanical Testing: The nanofibers mats were cut into 5mm × 10mm rectangular samples and attached onto paper windows using double sided tape. Tensile properties of nanofiber mats were determined with Bose Electroforce (Bose Corporation, Eden Prairie MN), with the use of 10 N load-cell under a cross head speed of 5mm/min. The thicknesses of samples were measured with a digital micrometer with a precision of 1 µm. At least five samples were tested for each type of electrospun fibrous membrane.

Differential Scanning Colorimetry: Thermal properties of the electrospun PCL and PCL-GE fibers were measured using a differential scanning calorimeter (DSC Q200 V24, TA Instruments New Castle, DE). The sample weights used were in the range of 2 to 3 mg and were placed in Tzero Aluminum pan. The instrument was calibrated with an indium standard. A nitrogen atmosphere (flow rate = 50.0 mL/min) was used throughout. All samples were first quenched to −80 °C with liquid nitrogen and then heated at a rate of 10 °C/min to 220 °C.

### *In vitro* cell culture

#### hSKP isolation and culture

Approval for acquiring human skin samples was provided by the University of Calgary Research Ethics Board. Adult human skin samples were obtained from deceased organ donors through the Southern Alberta Organ Transplantation and Tissue Recovery Program. Adult human SKPs were isolated from thigh or scalp donated tissue. Briefly, full thickness skin was washed with 70% ethanol, hair trimmed and underlying fat removed. The skin was cut into thin strips and incubated in dispase (5 mg/mL, Stem Cell Technologies) for 4 hrs in a water bath at 37 °C. The epidermis was manually removed with fine forceps leaving the dermal portion of the skin for cell isolation. This portion was washed in HBSS and cut into very small pieces using #20 scalpels. The tissue was incubated in collagenase IV (2 mg/mL, Worthington) in a water bath at 37 °C over a 4 hrs period. At each hour time point, the tissue was triturated and supernatant collected, filtered through a 70 μm filter and centrifuged for 6 mins at 200 rcf. The pellet was resuspended in 20.0 mL of F12 while the supernatant was replaced with the remaining tissue and incubated further. After 4 hrs, the total cell suspension was centrifuged for 6 mins at 200 rcf, the supernatant discarded, then resuspended in hSKP proliferation media (PM) composed of basic fibroblast grown factor (bFGF, 40 ng/mL, BD Biosciences), platelet-derived growth factor BB (PDGF-BB, 25 ng/mL, BD Biosciences), B27 supplement (2%, Invitrogen), penicillin/streptomycin (1%, Invitrogen) and fungizone (0.4%, Invitrogen) in DMEM low glucose/F12 (3:1, Invitrogen). Cells were resuspended at 60,000 cells per mL and plated onto suspension tissue culture flasks at 0.4 mL/cm^2^. Cells were fed with growth factors, B27, fungizone, and penicillin/streptomycin every 3–4 days until spheres reached 200 µm diameter. hSKPs were then dissociated to single cells using collagenase digestion and replated at the same density.

#### Cell Seeding

Nanofiber meshes were cut into 12 mm circular samples using a biopsy punch and immersed in 70% ethanol for 40 mins. The samples were then washed with PBS three times and were further sterilized by exposing them to UV light for 2 hrs. After sterilization, the nanofiber meshes were equilibrated with DMEM for 2 hrs before seeding cells. A 20.0 µl cell suspension containing 60,000 hSKPs was seeded onto each fiber sheet in 24-well culture plates and allowed to settle and attach for 2 hrs at 37 °C. A stainless steel sterilized circular ring (10mm internal diameter) was placed on top of each fiber mesh to keep the sample at the bottom of the plate. hSKP proliferation medium (1.0 mL) was added after 2 hrs into each well. Cells were fed with growth factors, B27, fungizone, and penicillin/streptomycin every 3–4 days.

#### Cell Proliferation Assay

hSKP-seeded nanofiber mats were harvested at day 1, 3 and 7 for cell counting and at day 3, 14 and 28 for DNA quantification and washed 3 times with PBS. Nanofiber mats were then incubated in collagenase IV (5.0 mg/mL, Worthington) for 5 to 10 mins at 37 °C and cells were counted using trypan blue and a hemocytometer. Cell viability within nanofiber mats after 1, 3 and 7 days of culture was examined using a LIVE/DEAD viability kit (Invitrogen cat #L3224). Briefly, media was removed and the mats were washed 3 times with PBS and incubated in diluted staining solution of ethidium homodimer-1 and calcein AM for 20 mins. The number of viable cells versus dead cells was accessed using ImageJ. The DNA content of hSKP-seeded scaffolds was measured by CyQuant NF cell proliferation assay kit (Invitrogen cat #C35006). At various time points, cell-populated nanofiber meshes were washed with PBS and frozen at −80 °C. After thawing, the scaffolds were digested with papain digestion solution for 48 hrs at 60 °C. The digested solutions were stained with CyQuant dye for 1 hr at 37 °C. Fluorescent intensity was measured at an excitation/emission of 450/520 nm using a microplate reader (BioTek Synergy, USA).

#### Cell Adhesion

Samples were fixed with 2.5% glutaraldehyde at 1, 3 and 7 days post seeding. Nanofibrous specimens were rinsed with water and dehydrated in graded series (30, 50, 60, 75, 90 and 100%) of ethanol. Subsequently samples were treated with hexamethyldisilazane (HMDS) and kept in a fume hood overnight to air dry. Samples were then coated with gold to observe cell morphology and adhesion under SEM.

#### Biochemical analysis for sulfated glycosaminoglycans (GAGs) and collagen

At predetermined time points, the media was removed and scaffolds were washed with PBS three times and frozen at −80 °C until further processing. The frozen samples were digested with papain digest solution (125 μg/mL papain, 5 mM L-cysteine hydrochloride, 100 mM Na_2_HPO4 and 5 mM Na_2_EDTA) in a dry oven for 24 hrs. GAG content was measured using DMMB staining and absorbance at 525 nm using a microplate reader. A chondroitin sulfate standard curve was obtained to evaluate the concentration of GAG desired. Total collagen content produced by hSKPs seeded onto nanofiber matrices was quantified by hydroxyproline assay using the hydroxyproline standard curve already described^41^. Absorbance was measured at 540 nm on a plate reader (BioTek Synergy, USA). Absorbance was normalized with nanofiber meshes of PCL-GE in order to subtract the background reading of gelatin used to fabricate nanofiber meshes. The resulting quantity of hydroxyproline was converted to collagen contents following a 1:10 ratio of hydroxyproline to collagen^42^.

#### Lentivirus transfection of hSKP cells

hSKPs were transfected with a TdTomato lentivirus. Briefly, hSKPs were plated at 200,000 cells per mL in a 6-well plate and incubated for 4 hrs at 37 °C. The cells were treated with 1.0 mL of TdTomato lentivirus supernatant and 3.0 μL of Polybrene for 18 hrs at 37 °C. The cell suspension was collected, centrifuged at 600 rcf for 6 mins and resuspended in hSKP PM and plated back into the 6-well plate. Cells were fed every 3–4 days until cells were confluent. Cells were then FACS-sorted to select cells expressing the TdTomato reporter and replated at 60,000 cells per mL in hSKP PM.

#### SHG imaging of collagen

Nanofiber scaffolds were imaged intact using a multi-photon confocal microscope (Zeiss 710) and SHG. Briefly, scaffolds were placed in a petri dish and covered with PBS to maintain hydration. A coverslip was placed over the sample to maintain a flat surface. TdTomato^+^ cells were imaged using a 514 nm Argon laser. Collagens were imaged with SHG with a Ti:Sa Chameleon multi-photon tunable laser (Coherent, Santa Clara, USA) at 800 nm, a custom BP:414/46, DC: 495, BP:525/50 filter, a dichroic mirror and a 20x water immersion objective.

#### Immunostaining of hSKPs seeded on nanofiber scaffolds

The scaffolds were washed twice with PBS and fixed with 4% paraformaldehyde for 10 mins and rinsed with PBS, followed by permeabilization using 0.5% Triton X-100 overnight and blocking with 10% donkey serum for 60 mins at RT. The scaffolds were then incubated with primary antibodies in blocking buffer overnight at 4 °C. The primary antibodies used in this study were goat anti-integrin α-9 (1:200, R&D System cat #AF3827), mouse anti-fibronectin (1:100, BD Bioscience cat #610078), rabbit anti-procollagen III (1:200, Millipore cat #AB764P) and rabbit anti-procollagen I (1:200, Millipore cat # MAB3391). After washing the samples with PBS, secondary antibodies were added to scaffolds for 1 hr at RT followed by nuclear stain Hoechst 33258 (1:5000). The images were acquired using a confocal fluorescent microscope (Leica SPS8).

### *In Vivo* grafting

#### Grafting procedure

All animal work was approved by the University of Calgary Health Sciences Animal Care Committee and was in accordance with the Canadian Council on Animal Care guidelines. 12-week-old male immunocompromised (Nu/Nu) mice were anesthetized using Isoflurane (n = 3 animals per scaffold group). Their backs were aseptically prepared before a 12 mm circular portion of skin was removed using a biopsy punch. Nanofiber meshes (12 mm diameter) were sterilized as previously described. Meshes were sutured into place (suturing the wound margin to the muscle underneath as well as the directly to the scaffold to prevent contracture) using 6.0 Vicryl sutures using a simple interrupted pattern. A Tegoderm, gauze, and Tenoplast bandage was applied and the wound was left to heal for 3 weeks. At 3 weeks, mice were euthanized by cervical dislocation under anesthesia and the back skin removed.

#### Wound closure analysis

Representative images (n = 3 per condition,) of each graft were taken at day 0 and at day 21. Using ImageJ, a known area was used for calibration and the area of the wound was outlined using the FreeHand Tool. The mean wound area was calculated at ‘day 0’ and was designated as “100%” of wound size. Identical measures were made for areas that had not been reepithelialized and expressed as a relative percentage of initial wound size on Day 0.

#### Tissue preparation and analysis

A 12 mm biopsy punch of the grafted back skin was obtained. One half was fixed in 10% neutral buffered formalin for 24 hrs before being prepared for histology. Briefly, samples were embedded in paraffin wax, sectioned at 4 µm thickness at 4 standardized levels throughout the graft (500 µm increments) and stained using Hematoxylin and Eosin staining. The remaining tissue halves were fixed overnight in 4% PFA at 4 °C then placed in increasing concentrations (10, 20 and 30%) of sucrose solutions at 4 °C every 24 hrs before being snap-frozen in blocks of OCT stored at −80 °C. Blocks were sectioned using a Leica CM1950 cryostat into 30 μm thickness sections and stored at −80 °C for immunohistochemical analysis. Hematoxylin and Eosin stained slides were digitally captured using an Olympus Virtual Slide System Macro Slide Scanner. Measurements of epidermal and dermal thickness and length of the graft were acquired using Olympius cellSens software. Measurements were acquired at 3 separate locations from each of 4 levels acquired 500 µm apart over the area of each graft and averaged to show epidermal and dermal thickness of the grafted area. To assess re-epithelialization of graft, the total length of the epithelium covering the graft was measured and divided by the total length of the graft to determine the percentage of re-epithelialization.

#### Immunohistochemistry

Epidermal proliferation: The epithelial layer was immunostained using a rabbit anti-mouse keratin 5 antibody (1:500, Covance cat #PRB-160P). Mitotically active nuclei were immunostained using a rat anti-mouse KI67 (1:100, eBioscience cat #SolA15,). Sections were incubated with primary antibodies in PBS containing 0.05% triton X-100 and 5% donkey serum overnight at 4 °C. Secondary antibodies (donkey anti-rabbit-Alexa488 and donkey anti-rat-Alexa555, Invitrogen) were used at 1:500 in PBS and incubated for 1 hr at room temperature. Nuclei were stained with Hoechst at 1:500 in PBS for 10 mins at RT, mounted using Permaflour mounting media and stored at 4 °C until imaging. Imaging was performed using a Leica SP8 spectral confocal microscope. Epidermal proliferation was assayed by counting the number of positively stained nuclei in the epidermis in one frame of view, then expressed relative to the total nuclei in each frame of view.

Vascularization: Vascularization was immunolabeled using a mouse anti-human CD31 antibody (1:100, BD Biosciences cat #550389,) in 1% Donkey Serum PBS and incubated overnight at 4 °C. Secondary antibodies were used at 1:500 in PBS and incubated for 2 hrs at RT. Nuclei labelling was performed using Hoechst at 1:1000 in PBS for 10 mins at RT followed by mounting using Permaflour^TM^ mounting media, coverslipped and being stored at 4 °C. Immunoflourescence was imaged throughout the graft with a Zeiss Observer microscope using Axiovision software (Zeiss). Innervation was assayed by counting the number of positively staining vessels within the area of the graft, then expressed relative to the area of the graft.

Innervation: Innervation was immunolabeled using rabbit anti-ßIII tubulin antibody (Abcam cat #Ab18207) and detected with a chromogen (AEC). Briefly, paraffin embedded slides were de-paraffinized and boiled in unmasking solution (Vector laboratories #H-3300) for 30 mins. Once cool, slides were rinsed in sterile PBS and endogenous peroxidase activity quenched with peroxidase blocking reagent (AEC^+^ Envision kit cat #K40088) for 15 mins, then permeabilized for 2 hrs with 0.5% Triton X in 1% BSA (in PBS) at RT. Slides were incubated with primary antibody (1:1000 in in 0.05 mol/L Tris HCL Buffer with 1% BSA) for 2 hrs at RT before incubation with the rabbit conjugated polymer available in the AEC^+^ Envision kit. The AEC chromagen was placed on the slide for 15 mins followed by 2 mins of hematoxylin counterstain before rinsing in tap water. Slides were mounted with 1:10 glycerol:PBS and coverslipped. The positive chromogen (AEC^+^) labeled nerve fibers (ßIII tubulin^+^) were counted using light microscopy on a Zeiss Observer microscope. The number of positively stained nerve fibers crossing the basement membrane of the epidermis were counted and expressed as the distance seen from the edge of the graft (as percentage of total graft length) as well as the density of nerve fibers in one 20x image on each side of the graft margin at 3 levels throughout the graft.

Collagen SHG: Collagen III was immunolabeled with anti-collagen 3α1 antibody (1:200, Rockland, Limerick, PA, cat # 600-401-105;) followed by goat anti-rabbit Alexa-555 secondary antibody (1:500, Invitrogen, Carlsbad, CA, cat # A-11008) and imaged using the Zeiss 710 confocal system. Collagen III was quantified using image analysis software (Photoshop CS6; Adobe Systems, San Jose, CA) as previously described^43^. The area positive for collagen III immunostaining was measured relative to the area positive for SHG in the same field of view. In scaffolds with TdT^+^ cells grown *in vitro* the SHG signal intensity was measured using FIJI (histogram analysis tool) in flattened z-stacks of 50 μm (1 μm/section).

### Statistical analysis

All statistical analysis was undertaken using GraphPad Prism (v6.0). For the *in vitro* analysis, a two-way ANOVA followed by Tukey’s test was used. A t-test was performed to compare the mechanical data of nanofibers. For the *in vivo* analysis, Kruskall-Wallis with Dunn’s multiple comparison tests were used. A P-value of 0.05 was considered significant for all data. All *in vivo* graphs are presented as mean +/− standard error of the mean (SEM).

## Electronic supplementary material

Supplementary data
