# Supplementary material for: Biocomposite nanofiber matrices to support ECM remodeling by human dermal progenitors and enhanced wound closure
Source: Sci Rep. 2017 Aug 31;7:10291. doi: 10.1038/s41598-017-10735-x (PMC5579010; doi:10.1038/s41598-017-10735-x)
Supplement: Supplementary file 1 — Supplementary data [file 41598_2017_10735_MOESM1_ESM.docx]

**Biocomposite nanofiber matrices to support ECM remodeling by human dermal progenitors and enhanced wound closure**

Fraz Anjum, Natacha A. Agabalyan, Holly D. Sparks, Nicole S. Rosin, Jeff Biernaskie and Michael S. Kallos

***Supplementary information***


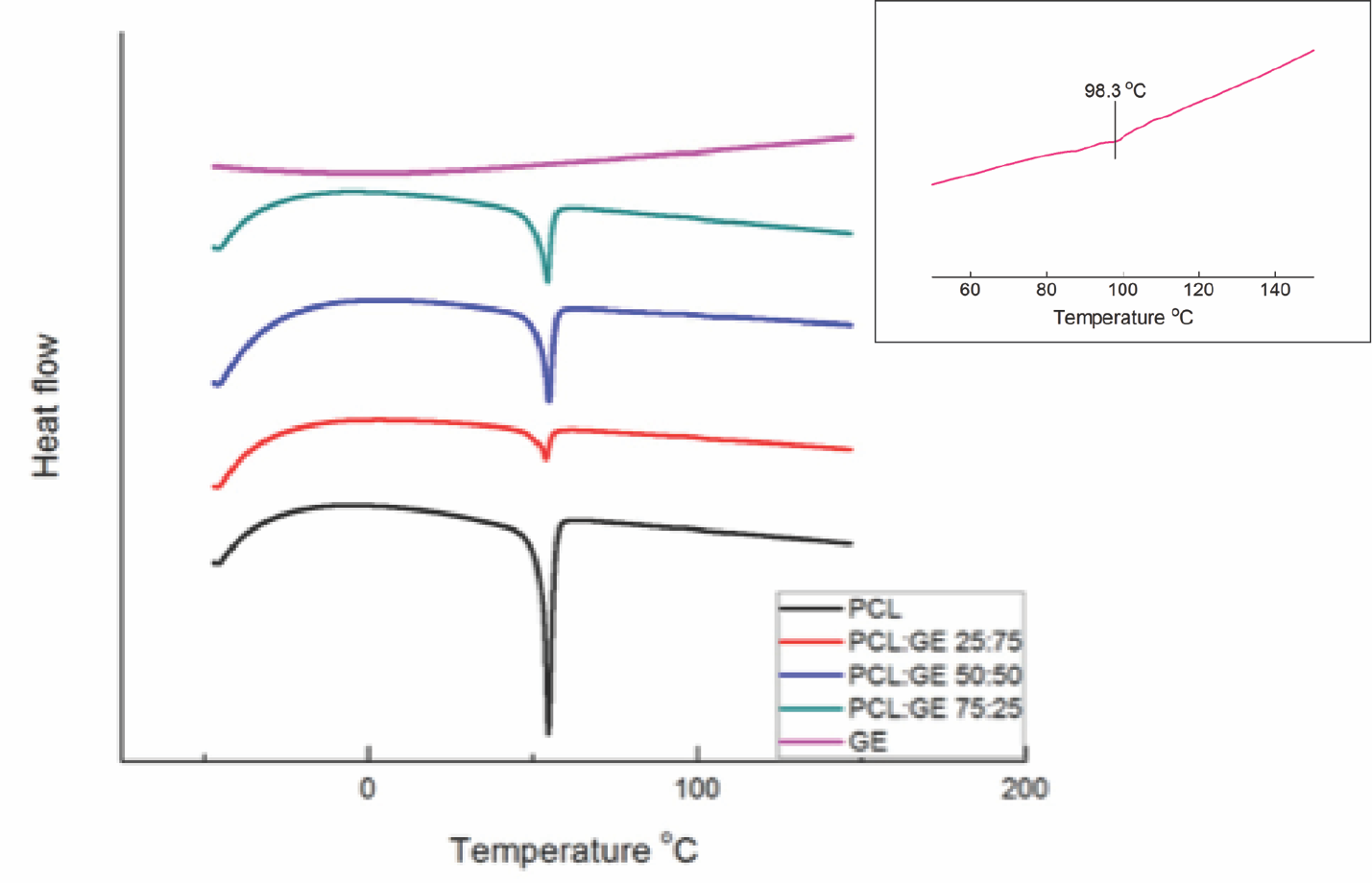


**Figure S-1:** DSC thermograms of nanofibres of PCL and PCL-GE composites. Three replicates were run in each sample.


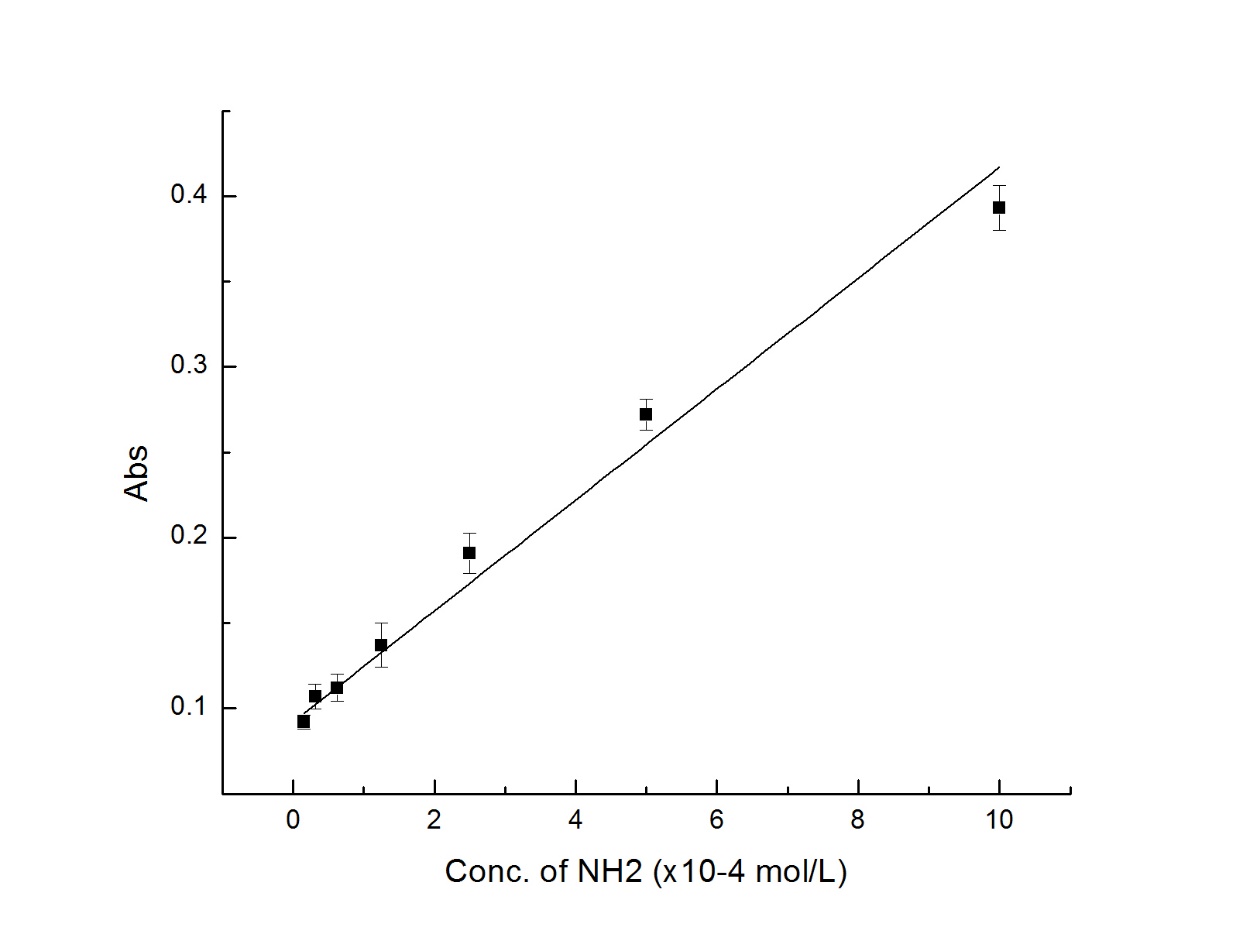


**Figure S-2:** Calibration curve obtained at 538 nm in 1, 4-dioxane/2-propanol (1:1) standard solutions ranging from 0.1 to 10 mM 1, 6-hexanediamine.


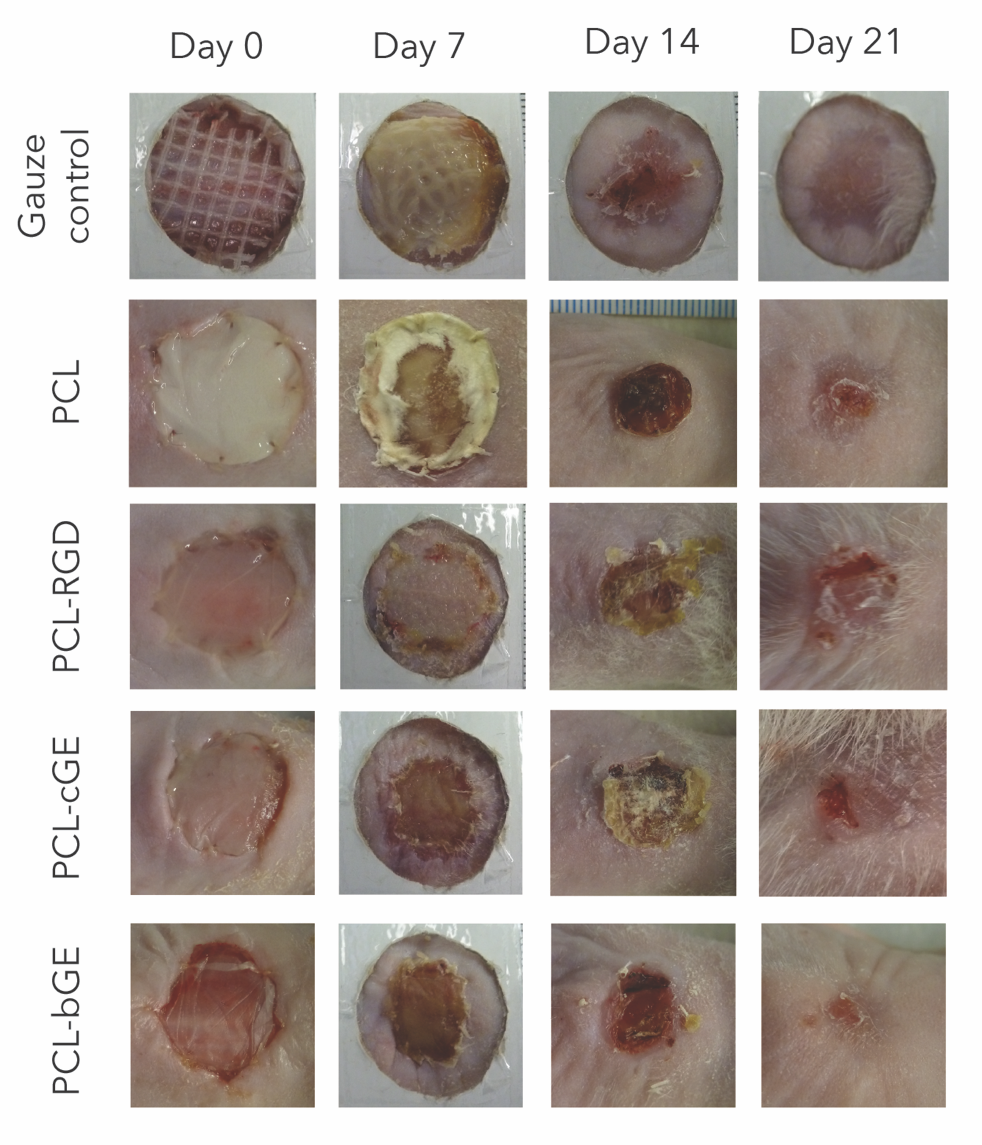


**Figure S-3:** Representative images of wound contraction over time.


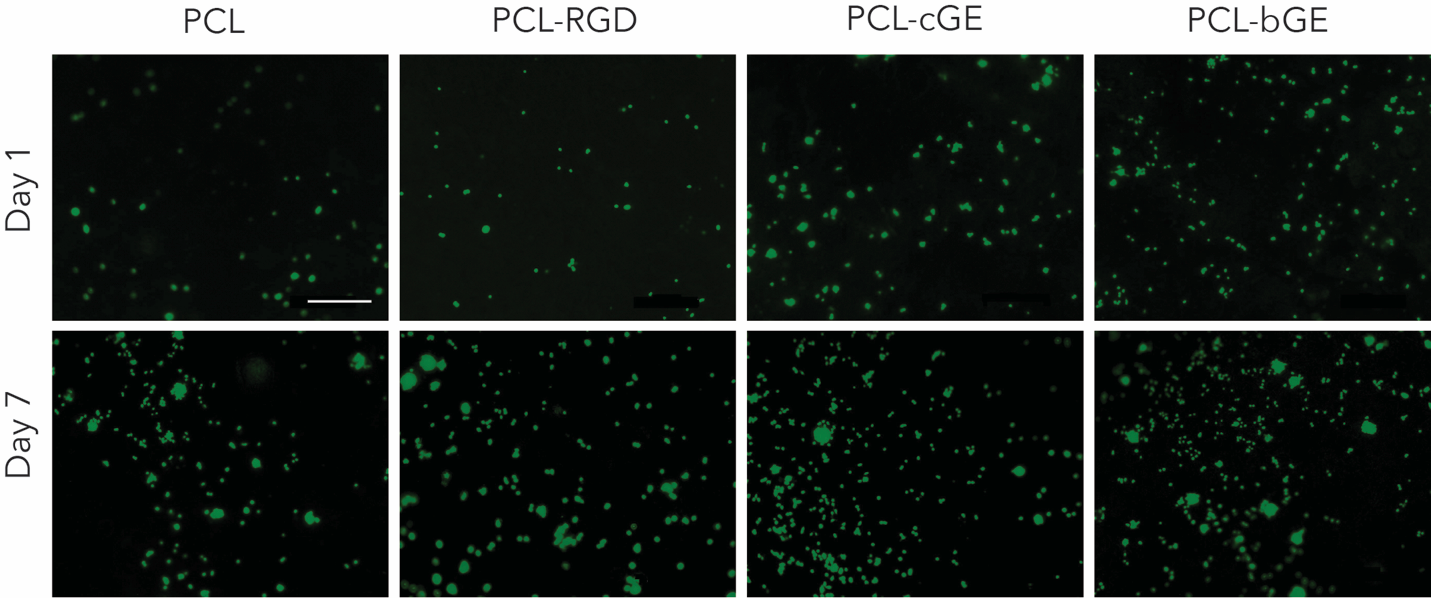


**Figure S-4:** Fluorescent images of hSKPs-seeded nanofibrous scaffolds stained by LIVE/DEAD viability kit at 1 and 7 day of culture showing spreading and proliferation of hSKPs. Bar = 500μm.


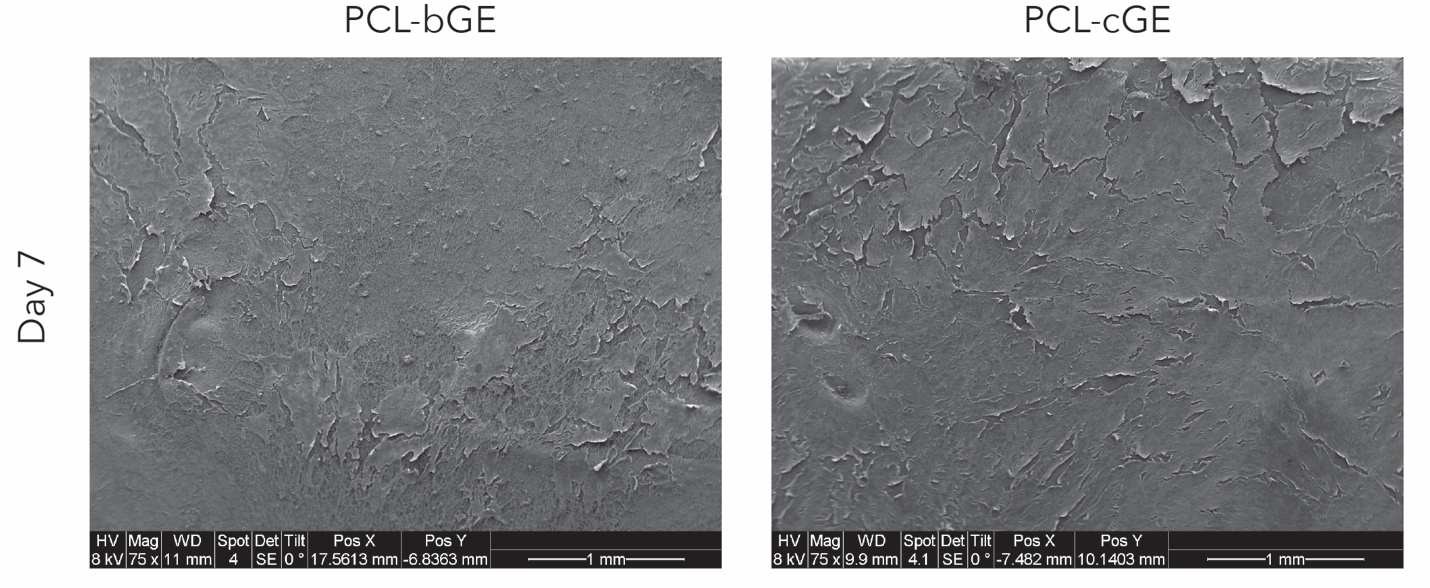


**Figure S-5:** SEM micrographs of hSKPs seeded nanofibre scaffolds showing complete confluency of cells at 7 days of culture.

| **Number of cells** | | | **p-value** | | | |
| --- | --- | --- | --- | --- | --- | --- |
| **Day 1** | **PCL vs PCL-RGD** | 0.011 | | * | |  |
|  | **PCL vs PCL-cGE** | 0.01 | | ** | |  |
|  | **PCL vs PCL-bGE** | 0.044 | | * | |  |
| **Day 3** | **PCL vs PCL-RGD** | 0.01 | | ** | |  |
|  | **PCL vs PCL-cGE** | 0.016 | | * | |  |
|  | **PCL vs PCL-bGE** | 0.004 | | ** | |  |
| **Day 7** | **PCL vs PCL-RGD** | 0.001 | | *** | |  |
|  | **PCL vs PCL-cGE** | 0.0012 | | ** | |  |
|  | **PCL vs PCL-bGE** | 0.0014 | | ** | |  |
|  |  |  | |  | |  |
| **DNA (ng/ml)** |  | **p-value** | |  | |  |
| **Day 14** | **PCL vs PCL-RGD** | 0.046 | | * | |  |
|  | **PCL vs PCL-bGE** | 0.001 | | *** | |  |
| **Day 28** | **PCL vs PCL-RGD** | 0.002 | | ** | |  |
|  | **PCL vs PCL-cGE** | 0.0001 | | **** | |  |
|  | **PCL vs PCL-bGE** | 0.0002 | | *** | |  |
|  |  |  | |  | |  |
| **GAG (µg/ml)** |  | **p-value** | |  | |  |
| **Day 14** | **PCL vs PCL-cGE** | 0.042 | | * | |  |
|  | **PCL vs PCL-bGE** | 0.05 | | * | |  |
| **Day 28** | **PCL vs PCL-RGD** | 0.001 | | *** | |  |
|  | **PCL vs PCL-cGE** | 0.001 | | *** | |  |
|  | **PCL vs PCL-bGE** | 0.0017 | | ** | |  |
|  |  |  | |  | |  |
| **GAG/DNA (ng/ng)** | | | **p-value** | |  | |
| **Day 3** | **PCL vs PCL** | 0.041 | | * | |  |
|  | **PCL-RGD vs PCL-RGD** | 0.05 | | * | |  |
|  | **PCL-cGE vs PCL-cGE** | 0.015 | | * | |  |
|  | **PCL-bGE vs PCL-bGE** | 0.01 | | * | |  |
|  |  |  | |  | |  |
| **Collagen (µg/ml)** | | | **p-value** | |  | |
| **Day 3** | **PCL vs PCL-cGE** | 0.001 | | *** | |  |
|  | **PCL vs PCL-bGE** | 0.001 | | *** | |  |
| **Day 14** | **PCL vs PCL-cGE** | 0.0011 | | ** | |  |
|  | **PCL vs PCL-bGE** | 0.0017 | | ** | |  |
| **Day 28** | **PCL vs PCL-RGD** | 0.0017 | | ** | |  |
|  | **PCL vs PCL-cGE** | 0.0014 | | ** | |  |
|  | **PCL vs PCL-bGE** | 0.0011 | | ** | |  |
|  |  |  | |  | |  |
| **Collagen/DNA (ng/ng)** | | | **p-value** | |  | |
| **Day 3** | **PCL vs PCL-cGE** | 0.0011 | | ** | |  |
|  | **PCL vs PCL-bGE** | 0.0017 | | ** | |  |
|  | **PCL-RGD vs PCL-cGE** | 0.0017 | | ** | |  |
|  | **PCL-RGD vs PCL-bGE** | 0.0014 | | ** | |  |
| **Day 14** | **PCL vs PCL-cGE** | 0.041 | | * | |  |
|  | **PCL vs PCL-bGE** | 0.05 | | * | |  |
|  | **PCL-RGD vs PCL-cGE** | 0.044 | | * | |  |
| **Day 28** | **PCL vs PCL-cGE** | 0.041 | | * | |  |
|  | **PCL vs PCL-bGE** | 0.05 | | * | |  |

**Table S-1:** Detail of p-values for Figure 6.
